# Circular RNA SMAD4 coordinates with m^6^A‐dependent IGF2BP2 to promote gallbladder cancer development

**DOI:** 10.1002/ctm2.70892

**Published:** 2026-11-05

**Authors:** Yiming Li, Yayun Xu, Yuan Zhang, Si Gao, Lili Gao, Zhiqing Yuan, Ziyi Wang, Shimei Qiu, Peipei Xu, Yuanding Liu, Zhaonan Liu, Huijie Miao, Hang Xu, Maolan Li, Rong Shao, Yingbin Liu

**Affiliations:** ^1^ Department of Biliary‐Pancreatic Surgery Renji Hospital Affiliated to Shanghai Jiao Tong University School of Medicine Shanghai China; ^2^ Shanghai Key Laboratory of Cancer Systems Regulation and Clinical Translation Jiading District Central Hospital Affiliated to Shanghai University of Medicine & Health Science Shanghai China; ^3^ Shanghai Cancer Institute State Key Laboratory of Systems Medicine for Cancer Shanghai China; ^4^ Department of Pathology Xinhua Hospital Affiliated to Shanghai Jiao Tong University School of Medicine Shanghai China; ^5^ Shanghai Key Laboratory of Biliary Tract Disease Research and Department of Surgery Xinhua Hospital Shanghai Jiao Tong University School of Medicine Shanghai China

**Keywords:** circSMAD4, Gallbladder cancer (GBC), IGF2BP2, m^6^A modification, nuclear export

## Abstract

Circular RNAs (circRNAs) have emerged as important regulators in tumorigenesis through microRNA sponging, protein translation and interaction with RNA‐binding proteins. Here, we identified a novel circRNA termed circSMAD4 that was generated by alternative splicing from exon 2 to exon 10 and back to the beginning of exon 2 of SMAD4 pre‐mRNA, constituting a 1435‐nucleotide circular RNA. circSMAD4 was upregulated in gallbladder cancer (GBC) tissues and positively correlated with cancer progression. Elevated expression and tumorigenic activity of circSMAD4 mainly relied on its interaction with IGF2BP2, where m^6^A‐binding motifs located at KH3 and KH4 of IGF2BP2 recognized and bound to the flanking intron sequences of circSMAD4. In the cytoplasm, IGF2BP2 stabilized circSMAD4 levels, and circSMAD4 in turn inhibited ubiquitination and degradation of IGF2BP2, thereby creating a positive feedback loop for both elevated expressions. Collaboration of circSMAD4 and IGF2BP2 activated JAK2/STAT3 and epithelial–mesenchymal transition (EMT) signalling. Notably, treatment of xenografted mouse models with CWI1‐2, a small molecule for interruption of IGF2BP2 binding to circRNA, resulted in decreases in circSMAD4‐induced tumour development. Collectively, our findings suggest that circSMAD4 cooperates with IGF2BP2 to promote tumour progression, highlighting the circSMAD4‐IGF2BP2 complex as a potential therapeutic target in GBC.

## Highlights

- circSMAD4 is upregulated in GBC and is associated with poor survival.
- IGF2BP2 promotes circSMAD4 biogenesis through m^6^A‐dependent recognition mediated by its KH3‐KH4 domains.
- circSMAD4 and IGF2BP2 reciprocally stabilize each other, forming a positive feedback loop that activates JAK2/STAT3 signalling and EMT.
- CWI1‐2 disrupts the circSMAD4‐IGF2BP2 interaction and suppresses circSMAD4‐driven tumour growth in vivo.

## Graphical abstract

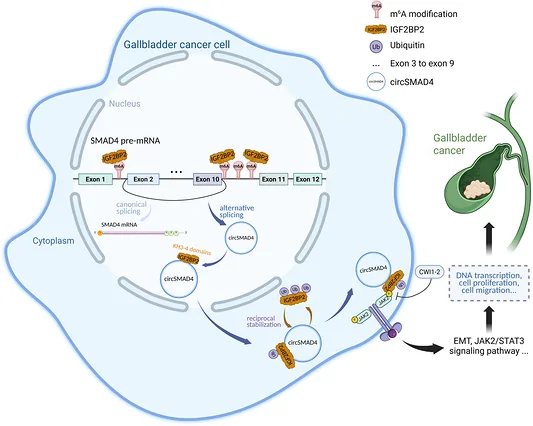

**Abbreviations**
SMAD4: SMAD family member 4
circRNA: circular RNA
circS: circular RNA SMAD4
EIBs: exon–intron boundaries
EIF4A3: eukaryotic translation initiation factor 4A3
ELAVL1: ELAV‐like RNA‐binding protein 1
EMT: epithelial–mesenchymal transition
ERBB2/3: Erb‐B2 receptor tyrosine kinase 2 or 3
EV: empty vector
FMR1: fragile X mental retardation 1
FUS: Fused in sarcoma
GBC: gallbladder cancer
HNRNPC: heterogeneous nuclear ribonucleoprotein C
IGF2BP2: insulin‐like growth factor 2 mRNA‐binding protein 2
JAK2: Janus kinase 2
MeRIP: methylated RNA immunoprecipitation.
MS: mass spectrometry
MUT: mutant
p‐JAK2: phosphorylated Janus kinase 2
PRPF8: pre‐mRNA processing factor 8
p‐STAT3: phosphorylated signal transducer and activator of transcription 3
PT: para‐tumour
RAS: rat sarcoma viral oncogene homolog
RBP: RNA‐binding protein
RIP: RNA immunoprecipitation
SMAD4: SMAD family member 4
STAT3: signal transducer and activator of transcription 3
T: tumour
TGF‐β: transforming growth factor‐β
TP53: tumour protein p53
U2AF2: U2 small nuclear RNA auxiliary factor 2
WT: wild type

## 1 INTRODUCTION

Gallbladder cancer (GBC), the most prevalent and aggressive malignancy of the biliary tract, emerges as a poor prognostic disease with a 5‐year survival rate of approximately 5%∼10%.^1^, ^2^, ^3^, ^4^ Surgical resection remains the only potentially curative intervention; however, the majority of patients are diagnosed at advanced stages during the first clinical visit, rendering them inoperable and subsequently selecting alternative therapeutic opportunities with radiotherapy, chemotherapy, and/or immunotherapy.^5^, ^6^, ^7^, ^8^ Over the past decades, a wealth of basic research focusing on multiple gene mutations (e.g., *RAS*, *TP53*, *SMAD4*, *ERBB2/3*),^9^, ^10^, ^11^, ^12^, ^13^, ^14^ together with diverse molecular regulators including coding proteins and non‐coding RNAs, has offered varied targeted therapies for GBC. Unfortunately, the efficacy is limited, and the prognosis remains poor. Thus, fully understanding their intimate interaction and mechanistic regulation emerges as a prominent challenge.

Circular RNAs (circRNAs) are covalently closed single‐stranded RNA molecules generated predominantly through back‐splicing, in which a downstream splice donor is joined to an upstream splice acceptor.^15^, ^16^ According to their sequence composition and genomic origin, circRNAs can be broadly classified into exonic circRNAs, exon–intron circRNAs (EIciRNAs), circular intronic RNAs, and mitochondria‐encoded circRNAs.^15^, ^16^ Unlike linear RNAs, circRNAs lack free 5′ and 3′ ends, rendering them relatively resistant to exonuclease‐mediated degradation and generally more stable than their linear counterparts. CircRNA biogenesis is tightly regulated by diverse cis‐acting elements and trans‐acting factors. In addition to complementary sequences and repetitive elements within flanking introns, exon–intron boundaries (EIBs) and other regions of cognate transcripts can also participate in the regulation of back‐splicing. RNA‐binding proteins (RBPs) and components of the splicing machinery act on these cis‐elements to modulate circRNA formation.^15^, ^17^, ^18^ Recent studies have further identified SRSF1 and ZC3H14 as important regulators of circRNA biogenesis. Notably, ZC3H14 binds to the 5′ and 3′ EIBs of circularized exons as well as the 3′ UTRs of cognate transcripts and facilitates back‐splicing through dimerization and association with the spliceosome.^17^, ^18^ These findings highlight that RBP‐mediated regulation of circRNA biogenesis is not restricted to flanking intronic sequences but can involve multiple cis‐regulatory regions of the precursor transcript.

Accumulating evidence indicates that circRNAs are not merely by‐products of RNA processing but function as versatile regulators of gene expression in a subcellular localization‐dependent manner. In the cytoplasm, circRNAs can act as microRNA decoys, interact with and modulate RBPs, regulate RNA stability and translation, and, in some cases, serve as templates for protein synthesis.^16^, ^19^ CircRNAs also exert important regulatory functions in the nucleus. EIciRNAs can interact with U1 small nuclear ribonucleoprotein and RNA polymerase II to enhance transcription of their parental genes in cis.^20^ Importantly, nuclear circRNAs can also directly modulate alternative splicing through interactions with splicing factors. For example, the predominantly nuclear circURI1 binds and sequesters hnRNPM, thereby altering exon inclusion or exclusion in multiple migration‐associated transcripts and suppressing gastric cancer metastasis.^21^ These findings reveal a reciprocal regulatory relationship between circRNAs and RBPs, whereby RBPs control circRNA biogenesis and fate, while circRNAs in turn modulate RBP activity and downstream RNA processing. Dysregulation of these processes has been increasingly implicated in tumour initiation, progression, metastasis, and therapeutic response, supporting circRNAs as potential biomarkers and therapeutic targets in cancer.^16^, ^19^

N^6^‐methyladenosine (m^6^A) serves currently as one of the most prevalent and abundant epigenetic modifications in both messenger RNAs (mRNAs) and non‐coding RNAs.^22^, ^23^ m^6^A modifications involve methylation by methyltransferases (METTL3, METTL14, METTL16) termed as m^6^A writers, methyl recognition by m^6^A binding proteins (YTHDF1/2/3 and IGF2BP1/2/3) known as m^6^A readers, and demethylation by demethylases (ALKBH3 and ALKBH5) referred to as m^6^A erasers.^24^ Aberrant m^6^A modifications can disrupt RNA transcription, translation, and degradation, thereby mediating tumorigenesis and cancer progression.^25^, ^26^ Interestingly, it has been reported that circRNAs with m^6^A modifications function differentially from their mRNA counterparts.^27^ However, the abundance and functional implications of m^6^A modifications on circRNA precursors and mature circRNAs remain largely undefined.

SMAD4 is a central mediator of canonical TGF‐β signalling and plays critical roles in the regulation of cell proliferation, differentiation, apoptosis, tumorigenesis, and cancer progression.^28^ Upon TGF‐β receptor activation, receptor‐regulated SMADs, primarily SMAD2 and SMAD3, associate with SMAD4 and translocate into the nucleus to regulate downstream transcriptional programs. In multiple cancer types, SMAD4 signalling is also subject to regulation by circRNAs. For example, in gastric cancer, hsa_circ_0004872 acts as a sponge for miR‐224, thereby upregulating SMAD4 and forming a negative feedback loop with ADAR1 that contributes to tumour suppression.^29^ In colorectal cancer, circPTEN1 suppresses metastasis by interfering with SMAD4‐mediated TGF‐β signalling.^30^ These findings highlight the ability of circRNAs to modulate canonical SMAD4‐dependent signalling in cancer.

Beyond its canonical role in TGF‐β signalling, emerging evidence indicates that SMAD4 can also exert TGF‐β‐independent functions through protein‐protein interactions. Recent SMAD4‐focused oncogenic protein–protein interaction (OncoPPI) mapping in pancreatic ductal adenocarcinoma identified a direct interaction between SMAD4 and NFATc1 that occurs independently of TGF‐β signalling. This interaction restrains NFATc1 transcriptional activity, whereas loss of SMAD4 releases this inhibitory interaction and promotes NFATc1‐dependent upregulation of STAT3, revealing a non‐canonical SMAD4‐NFATc1‐STAT3 regulatory axis.^31^ In parallel, crosstalk between canonical TGF‐β signalling and JAK2/STAT3 has also been reported; for example, JAK2/STAT3 activation contributes to TGF‐β‐induced epithelial‐mesenchymal transition in lung cancer.^32^ Our previous work further demonstrated that the JAK2/STAT3 axis is markedly activated through GPRC5A upregulation in GBC, underscoring the relevance of this pathway to GBC progression.^33^ Together, these observations illustrate that SMAD4‐associated biology may extend beyond canonical TGF‐β signalling and intersect with STAT3 signalling through distinct molecular mechanisms.

Recent studies have also revealed diverse biological functions of circRNAs derived from the *SMAD4* locus. For instance, circSMAD4 (hsa_circ_0047718) promotes gastric cancer progression through activation of the Wnt/β‐catenin pathway,^34^ whereas another *SMAD4*‐derived circRNA (mmu_circ_0007494) has been reported to interact with RNA‐binding proteins and participate in post‐transcriptional regulation.^35^ Importantly, these previously reported circSMAD4 species are distinct from hsa_circ_0108590 investigated in the present study. Hsa_circ_0108590 is generated from exons 2–10 of the *SMAD4* gene, and its biological function and regulatory mechanisms in GBC remain unknown. Notably, IGF2BP2, an m^6^A reader and oncogenic RNA‐binding protein, is highly expressed in GBC and has been implicated in the regulation of RNA stability and translation.^21^, ^36^ Therefore, whether IGF2BP2 regulates the biogenesis and function of hsa_circ_0108590, and whether this circRNA contributes to GBC progression through signalling mechanisms distinct from or potentially interconnected with those of its linear host gene, remain important unanswered questions.

## 2 MATERIALS AND METHODS

### 2.1 Clinical specimens

Human gallbladder cancer (GBC) tissues and paired adjacent non‐tumorous specimens were obtained from 65 patients who underwent surgical resection at Renji Hospital Affiliated to Shanghai Jiao Tong University School of Medicine from 2020 to 2024. The diagnosis of GBC was confirmed independently by experienced pathologists at Renji Hospital. Detailed clinicopathological characteristics of the enrolled patients are provided in Table S3. Tumour grading and clinical staging were determined based on the criteria of the 8th edition of the American Joint Committee on Cancer (AJCC). All patients were subjected to regular postoperative follow‐up for clinical outcome monitoring and survival analysis.

### 2.2 Cell lines and cell culture

Human gallbladder cancer (hGBC) cell lines NOZ, GBC‐SD, ZJU‐0430, OCUG‐1, EHGB‐1, and HEK‐293T were purchased from Mingzhou Biotechnology (Ningbo) and Shanghai FuHeng Biotechnology. Human normal gallbladder epithelial cell line L‐2F7 was established in our lab.^37^ All cell lines were contamination‐free and were authenticated by STR (short tandem repeat). All hGBC cell lines were cultured in DMEM high glucose medium (Cat. 41401ES76, Yeasen) with 10% fetal bovine serum (FBS) (Cat. 164210, Procell) and 1% penicillin‐streptomycin solution (Cat. 60162ES76, Yeasen). L‐2F7 cells were cultured in the epithelial cell medium (EpiCM) (Cat. 4101, ScienCell, California, USA) with 10% FBS (Cat. 164210, Procell) and 1% penicillin‐streptomycin solution (Cat. 60162ES76, Yeasen). All adherent cells were digested with trypsin solution (Cat. 40127ES60, Yeasen) for 3 min before cell passage or being frozen using a serum‐free cell‐saving buffer (Cat. C30100, NCM Biotech).

### 2.3 Cell and tissue RNA isolation and qRT‐PCR

Cellular total RNA was isolated according to the manufacturer's guide for the RNA isolation kit (Cat. B0004D, EZBioscience), and tissue total RNA was isolated with TRIzol reagent. RNA concentrations were quantified by the NanoDrop 2000c spectrophotometer (Thermo Scientific). RNA was treated with gDNA wiper and then reverse transcribed to cDNA according to the manufacturer's manual of HiScript II Q Select RT SuperMix(Cat. R233‐01, Vazyme). Quantitative PCR reaction mix was prepared according to the guidelines of the ChamQ Universal SYBR qPCR Master Mix (Cat. Q711‐02/03, Vazyme) and run in a qRT‐PCR system (QuantStudio Dx, Applied Biosystems). All the primers used in this study are listed in Table S1.

### 2.4 Co‐immunoprecipitation and ubiquitination assay

Cells subjected to the indicated treatments were washed twice with ice‐cold PBS and lysed on ice in Western/IP lysis buffer (#P0013, Beyotime) supplemented with protease inhibitor cocktail (#ST506, Beyotime). Cell lysates were clarified by centrifugation at 14 000×*g* for 10 min at 4°C, and protein concentrations were determined using a BCA protein assay kit (Beyotime, #P0010). Equal amounts of total protein were incubated with anti‐IGF2BP2 antibody or the corresponding normal IgG control overnight at 4°C with gentle rotation. Protein A/G beads (Selleckchem, #B23202) were then added and incubated for an additional 6 h at 4°C. The immune complexes were collected and washed three times with ice‐cold Western/IP lysis buffer. Bound proteins were eluted by boiling the beads in SDS buffer and subsequently analyzed by Western blotting.

For ubiquitination assays, cells were treated with MG132 at 2 µM for 6 h before harvest to inhibit proteasomal degradation and facilitate the accumulation of ubiquitinated proteins. IGF2BP2 was then immunoprecipitated as described above, and the precipitated proteins were subjected to immunoblotting with an anti‐ubiquitin antibody (#3933, CST) to determine IGF2BP2 polyubiquitination levels. IGF2BP2 immunoblotting of the immunoprecipitated fractions was performed to verify comparable immunoprecipitation efficiency, and total cell lysates were analyzed in parallel as input controls. For gain‐ and loss‐of‐function analyses, NOZ and GBC‐SD cells were transfected with circSMAD4 overexpression plasmids, two independent circSMAD4‐targeting siRNAs, or the corresponding controls before immunoprecipitation.

### 2.5 Subcutaneous mouse models

BALB/c nude mice with 5–6 weeks old were obtained from Shanghai Model Organisms (https://www.modelorg.com/, Shanghai, China). A total of 1 × 10^6^ wild‐type GBC cells or GBC cells stably overexpressing circSMAD4 were subcutaneously injected into the flanks of mice. Seven days later, mice were treated with either 100 µL 5 mg/kg CWI1‐2 (Cat. HY‐153274, MCE) or vehicle control (DMSO) via tail vein injection once every 3 days. Tumour growth was monitored using calliper measurements at indicated time points and was calculated using the equation: *V* = 1/2 × (length × width^2^). After 4 weeks, mice were euthanized, and subcutaneous tumours were subsequently harvested for further analysis.

### 2.6 Ethics

This study was approved by the Ethics Committee of Renji Hospital, Shanghai Jiao Tong University School of Medicine (Approval No. KY2024‐036‐C), and conducted in accordance with the ethical principles outlined in the Declaration of Helsinki. Written informed consent was obtained from all participants prior to specimen collection. All animal experiments were reviewed and approved by the Institutional Animal Care and Use Committee (IACUC) of Renji Hospital, Shanghai Jiao Tong University School of Medicine. All procedures involving animals were performed in strict compliance with institutional guidelines and the ARRIVE (Animal Research: Reporting of In Vivo Experiments) guidelines to ensure ethical and humane treatment.

### 2.7 Statistical analysis

All experiments were performed in at least three independent biological replicates. Quantitative data were presented as mean ± standard deviation (SD). Statistical analyses were conducted using GraphPad Prism 8.0 (GraphPad Software) and R software (version 4.5.0). Comparisons between two groups were evaluated using the unpaired two‐tailed Student's *t*‐test. Paired two‐tailed Student's *t*‐tests were used for comparisons between matched GBC and adjacent non‐cancerous tissues. CircRNA expression heatmap in 3 paired GBC tissues was analyzed in R and compared using Wilcoxon test.

For clinicopathological and survival analyses, circSMAD4 expression was quantified by qRT‐PCR in tumour tissues from 65 patients with GBC. The relative expression values were normalized such that the cohort median was set to 1.0. Patients with circSMAD4 expression values ≥1.0 were classified as the high‐expression group, whereas those with values <1.0 were classified as the low‐expression group. Overall survival analyses were conducted using the Kaplan–Meier method and compared using the log‐rank test, also implemented in R software. The survival analysis was unadjusted, and multivariable Cox proportional hazards regression was not performed; therefore, the observed association between circSMAD4 expression and overall survival was not adjusted for potential clinicopathological confounders. Associations between circSMAD4 expression levels and clinicopathological variables were evaluated using the Chi‐square test or Fisher's exact test, as appropriate. Pearson correlation analysis was used to evaluate correlations between gene expression levels. All statistical tests were two‐sided unless otherwise specified. *p*‐values < .05 were considered statistically significant. Statistical significance was indicated as follows: *p < .05, **p < .01, ***p < .001, and ****p < .0001.

Please see supplementary materials for other detailed methods.

## 3 RESULTS

### 3.1 Characteristics and expression of circSMAD4 in GBC cells and tissues

To identify SMAD4‐associated circRNAs potentially involved in GBC development, we conducted circRNA sequencing on three pairs of patient‐derived GBC tissues and their corresponding adjacent non‐tumorous tissues (Figure 1A). This analysis yielded 1434 differentially expressed circRNAs (*p* < .05). Given the limited size of the initial sequencing cohort, the circRNA‐seq analysis was used primarily as an exploratory discovery screen, and candidate circRNAs showing consistent expression changes were subsequently prioritized for independent experimental validation. Among these candidates, we identified hsa_circ_0108590 as the only circRNA derived from the *SMAD4* locus, exhibiting consistently higher expression in all three GBC tissues compared with counterparts (Figure 1B).

**FIGURE 1:**
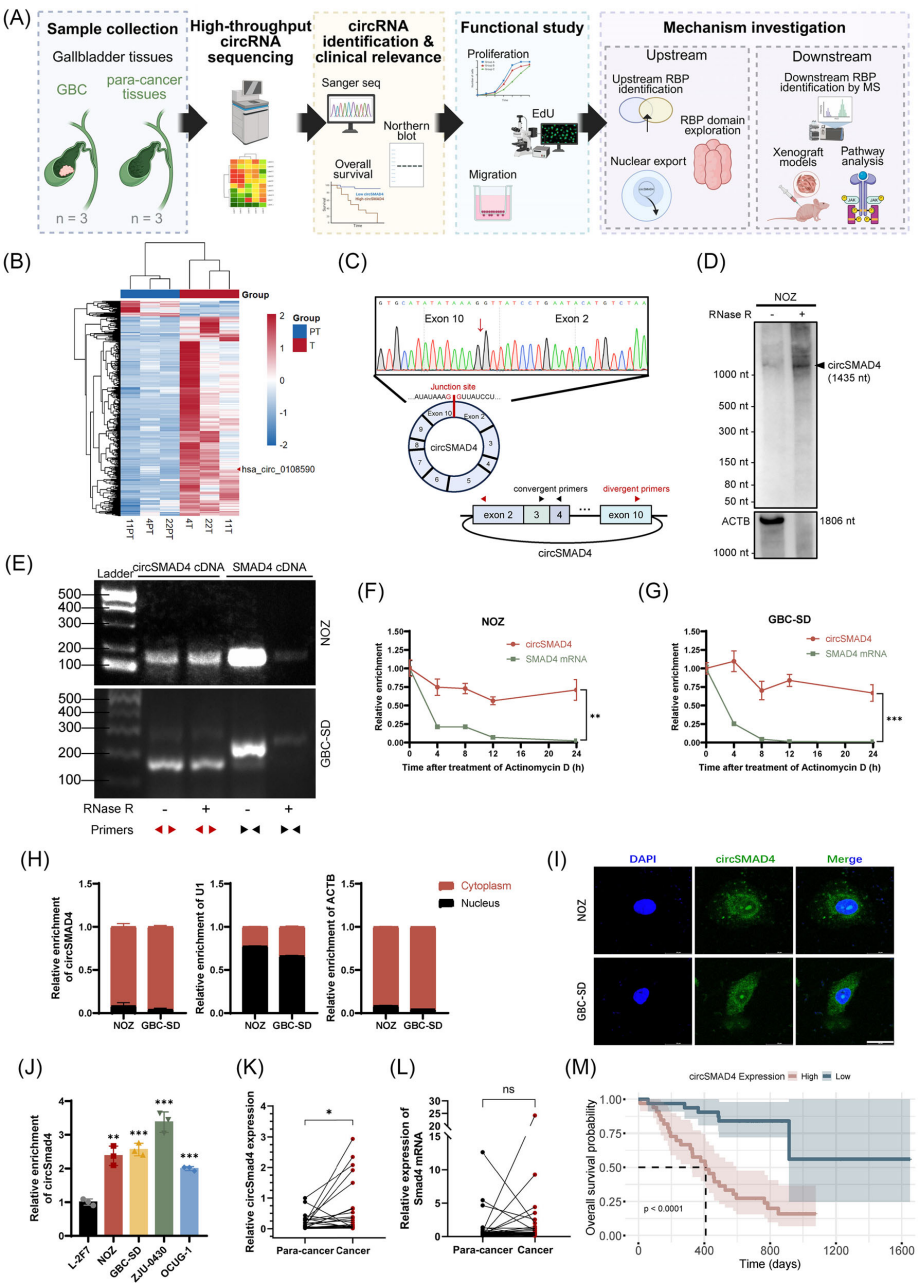
Identification and characteristics of circSMAD4 derived from *SMAD4*. (A) Schematic research workflow. GBC and para‐cancer tissues underwent circRNA sequencing, followed by validation and clinical correlation. Functional assays assessed proliferation and migration. Mechanistic studies included upstream RBP identification, nuclear export, and downstream analyses using mass spectrometry (MS), xenograft models, and pathway exploration. (B) Heatmap representing the differentially expressed circular RNAs in 3 patient‐derived GBC tissues (T) and counterpart para‐tumour tissues (PT). Red and blue indicate upregulated and downregulated circRNAs, respectively. Candidate circRNAs were selected based on a nominal *p*‐value < .05 in the exploratory analysis using the Wilcoxon test. (C) The schematic pattern of genomic loci of circSMAD4. The expression of circSMAD4 was validated by Sanger sequencing. The red arrow refers to the back‐splice junction site of circSMAD4. 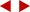 Divergent primers covering exon 3 to exon 4; 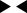 convergent primers covering exon 10 to exon 2. (D) Northern blotting of circSMAD4 by hybridization with the exon 10‐exon 2 junction probe in the presence of RNase R treatment. Left lane, 2 µg total RNA; right lane, 2 µg RNase R‐treated RNA. (E) PCR followed by agarose gel electrophoresis confirmed the presence of circSMAD4 and linear SMAD4 transcripts using divergent and convergent primers, respectively, with or without RNase R treatment. 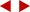 divergent primers; 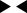 convergent primers. (F, G) qRT‐PCR for the abundance of circSMAD4 and SMAD4 mRNA treated with 5 µg/mL Actinomycin D in (F) NOZ and (G) GBC‐SD cell lines in the 24 h period. *n* = 3. (H) Relative RNA expression levels of circSMAD4 in the nuclear and cytoplasmic fractions of GBC cell lines NOZ and GBC‐SD were tested by qRT‐PCR. Relative RNA expression levels of U1 and ACTB were tested as positive nuclear and cytoplasmic controls, respectively, in the nuclear‐cytoplasmic fractionation assay. *n* = 3. (I) RNA fluorescence in situ hybridization (RNA‐FISH) assay showed the subcellular distribution of circSMAD4 in NOZ and GBC‐SD. Scale bar = 20 µm. (J) The expression level of circSMAD4 in normal gallbladder epithelial cells and gallbladder cancer cell lines. The quantified data were from RT‐PCR analyses; *n* = 3. (K) Relative expression level of circSMAD4 in 30 paired gallbladder cancer and adjacent para‐cancer tissues. The quantified data were from RT‐PCR analyses followed by normalization with the median as 1 unit. (L) Relative expression level of SMAD4 mRNA in 30 paired gallbladder cancer and adjacent para‐cancer tissues. (M) Kaplan–Meier analysis revealed that GBC patients (*n* = 65) with low circSMAD4 expression (*n* = 32) had significantly longer overall survival than those with high expression (*n* = 33). The *p*‐value was determined using the log‐rank test. Data are shown as the mean ± S.D. The *p*‐value was determined by a two‐tailed unpaired Student's *t*‐test. ns, not significant *p < .05, **p < .01, ***p < .001.

circSMAD4 (circBase ID: hsa_circ_0108590) was an exonic circular RNA generated from exons 2 to 10 of the *SMAD4* gene through alternative splicing of its pre‐mRNA. It comprised 1,435 nucleotides and was classified as a purely exonic circRNA (Figure S1A). To confirm it is a circular RNA, we designed divergent primers located at exon 10 and exon 2. Following RT‐PCR with divergent primers, the amplified product (167 bp) was confirmed to match the expected sequence by Sanger sequencing (Figure 1C). We subsequently designed a probe covering the exon 10‐exon 2 back‐splice junction to further validate the existence of circSMAD4 by Northern blotting (Figure 1D). To further confirm the characteristics of circSMAD4, we amplified circSMAD4 using divergent primers covering the junction site and SMAD4 mRNA using convergent primers from cDNA of GBC cell lines (NOZ and GBC‐SD), and treated them with or without RNase R. In sharp contrast to SMAD4 mRNA, which was sensitive to RNase R and fully degraded, circSMAD4 sustained a high level following RNase R treatment (Figure 1E), indicating circSMAD4 is a unique circular structure resistant to RNase R. Strikingly, circSMAD4 exhibited markedly greater stability compared with its linear counterpart SMAD4 mRNA in GBC cell lines NOZ and GBC‐SD. Following actinomycin D treatment, circSMAD4 maintained a half‐life exceeding 24 h, whereas SMAD4 mRNA decayed rapidly with an estimated half‐life of approximately 2 h (Figure 1F,G). In addition, more than 90% of circSMAD4 RNA was localized to the cytoplasm of NOZ and GBC‐SD cells relative to a small portion in the nucleus (Figure 1H). Consistently, fluorescence in situ hybridization (FISH) analysis confirmed that circSMAD4 was predominantly localized in the cytoplasm of these cells (Figure 1I).

Notably, circSMAD4 exhibited significantly elevated expression in GBC tissues compared with matched para‐tumour tissues (Figure 1A), suggesting a potential oncogenic role distinct from its linear host gene. We then performed qRT‐PCR analysis in 4 GBC cell lines and the normal gallbladder epithelial cell line L‐2F7,^37^ and found that circSMAD4 had 2‐3.2‐fold significantly higher expression levels in all 4 GBC cell lines compared with L‐2F7 (Figure 1J). Subsequently, qRT‐PCR analysis of extracted RNAs from 30 paired patient‐derived GBC cancer tissues and corresponding adjacent non‐cancerous tissues revealed that circSMAD4 was significantly upregulated in GBC cancerous tissues relative to adjacent non‐cancerous tissues (Figure 1K), whereas SMAD4 mRNA levels were identical (Figure 1L). To further validate the differential expression of circSMAD4 in patient‐derived samples, we conducted single‐molecule fluorescence in situ hybridization (smFISH) in 21 pairs of patient‐derived GBC tissues and adjacent non‐cancerous tissues. The results showed that circSMAD4 in GBC tissues was around threefold greater than counterpart controls (Figure S1B,C). Moreover, we extended the GBC cohort to 65 GBC patients and found that circSMAD4 levels were positively correlated with tumour size, T stage and TNM stage (AJCC 8th edition) (Table 1), and the overall survival time of GBC patients with high circSMAD4 levels was approximately 1 year compared with 5 years for patients with low levels (Figure 1M). In conclusion, our findings validated the loop structure of circSMAD4 that is upregulated in the progression of gallbladder cancer.

**TABLE 1:** Correlation between circSMAD4 expression and clinicopathological features in 65 GBC patients (Renji Cohort).

Characteristic	All cases	circSMAD4		Chi‐square	*p*‐value
		Low	High		
Sex				.012396	.9113^a^
Male	29	15	14		
Female	36	17	19		
Age (y)				4.07E‐31	1^a^
≥70	34	17	17		
<70	31	16	15		
Tumour size (cm)				11.208	**.0008145** ^*^ ^,^ ^a^
≥4.0	33	9	24		
<4.0	32	23	9		
Grade				.45728	.4989^a^
I–II	41	22	19		
III–IV	24	10	14		
T stage				15.607	**<.0001** ^**^ ^,^ ^a^ **<.0001** ^**^ ^,^ ^b^
T1‐2	24	20	4		
T3‐4	41	12	29		
N stage				1.5905	.2072^a^
N0	42	25	20		
N1‐2	23	7	13		
TNM stage				21.64	**<.0001** ^**^ ^,^ ^a^ **<.0001** ^**^ ^,^ ^b^
I–II	20	19	1		
III–IV	45	13	32		

*Note*: Bold *p*‐values indicate statistical significance (*p* < .05). The TNM staging was determined in strict accordance with the criteria outlined in the 8th edition of the American Joint Committee on Cancer (AJCC) classification system.

^a^Chi‐square test.

^b^Fisher's exact test.

^*^ *p* < .001.

^**^ *p* < .0001.

### 3.2 circSMAD4 promoted GBC cell proliferation and migration

To study the functional role of circSMAD4 in GBC progression, we knocked down circSMAD4 with two different siRNAs that specifically targeted the back‐splice junction region of circSMAD4, and overexpressed circSMAD4 in both NOZ and GBC‐SD cells (Figure 2A,B). A series of functional assays were performed to assess the biological roles of circSMAD4 in GBC. The CCK‐8 assay, EdU assay and cell colony formation assay revealed that knockdown of circSMAD4 reduced the viability of GBC cells and that overexpression of circSMAD4 promoted the growth of NOZ and GBC‐SD cells (Figure 2C,G–I). The Transwell migration assays displayed that downregulation of circSMAD4 inhibited the migration ability of NOZ and GBC‐SD cells, whereas overexpression of circSMAD4 induced cell migration (Figure 2J).

**FIGURE 2:**
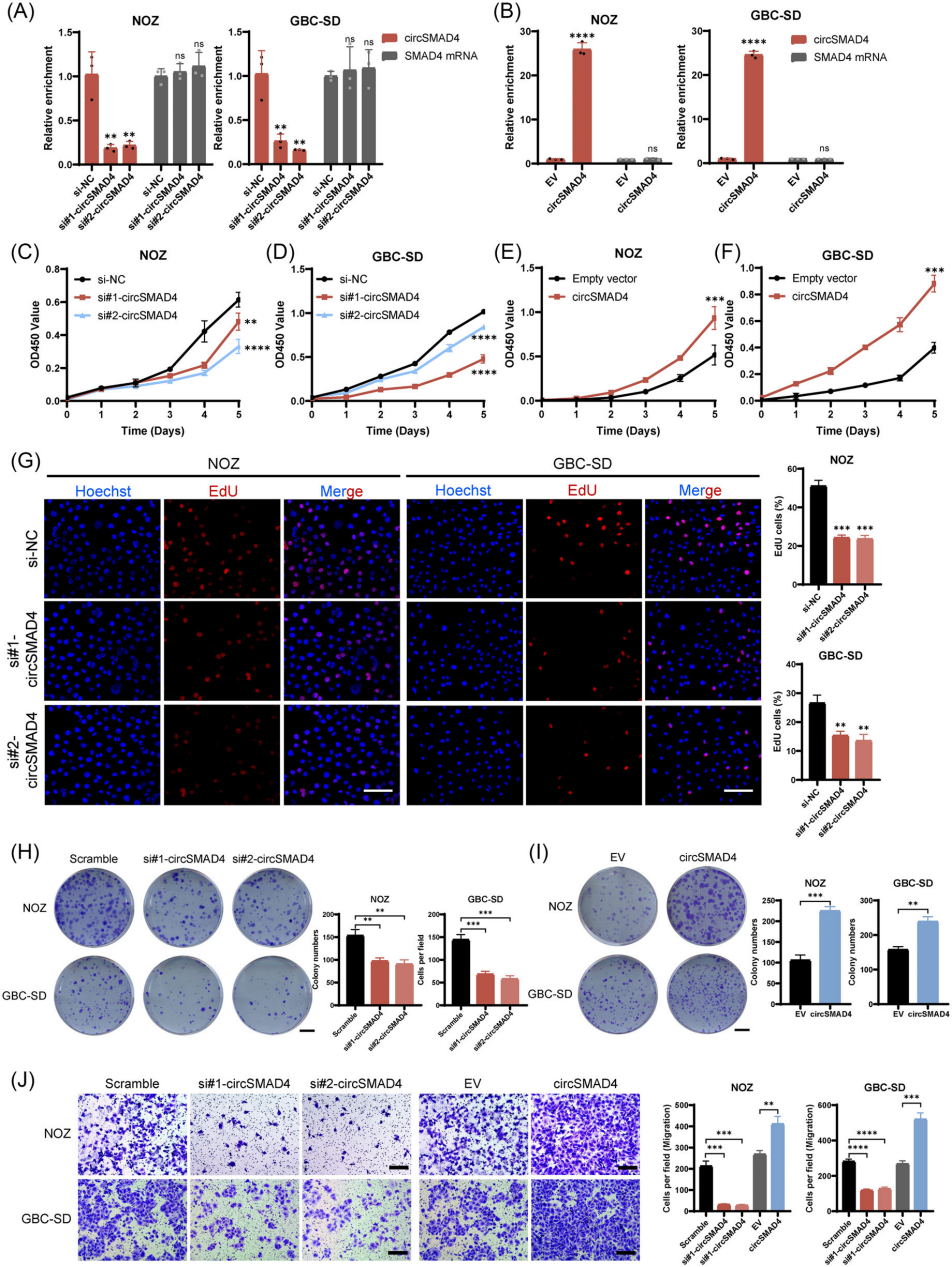
**circSMAD4 enhances proliferation and migration ability in GBC cells**. (A) Relative circSMAD4 and Smad4 mRNA expression levels in NOZ and GBC‐SD cell lines transfected with si‐NC, si#1‐circSMAD4or si#2‐circSMAD4 separately, as indicated in the RT‐PCR assay; *n* = 3. (B) Relative circSMAD4 and Smad4 mRNA expression levels in NOZ and GBC‐SD cell lines transfected with empty vector (EV) orcircSMAD4 plasmids separately, as indicated in the RT‐PCR assay; *n* = 3. (C, D) CCK‐8 assay of NOZ and GBC‐SD cell lines transfected with si‐NC, si#1‐circSMAD4 or si#2‐circSMAD4 for 5 days; *n* = 3. (E, F) CCK‐8 assay of NOZ and GBC‐SD cell lines transfected with empty vector (EV) or circSMAD4 plasmids for 5 days; *n* = 3. (G) Ethynyl‐2‐deoxyuridine (EdU) incorporation assay was performed in NOZ and GBC‐SD cell lines transfected with si‐NC, si#1‐circSMAD4 or si#2‐circSMAD4 as indicated. Scale bar: 100 µm; *n* = 3. (H, I) A colony formation assay was performed to assess cell growth in NOZ and GBC‐SD cell lines transfected with (H) circSMAD4 knockdown siRNA or (I) circSMAD4 overexpression plasmids, as indicated. Scale bar: 5 mm; *n* = 3. (J) Transwell assays of migration in NOZ and GBC‐SD cell lines transfected with circSMAD4 knockdown siRNA or overexpression plasmids, as indicated. Scale bar: 200 µm; *n* = 3. Data are shown as the mean ± S.D. The *p*‐value was determined by a two‐tailed unpaired Student's *t*‐test. ns, not significant *p < .05, **p < .01, ***p < .001, ****p < .0001.

To test the possibility that plasmids designed for circSMAD4 overexpression may potentially generate linear transcripts owing to inefficient circularization,^38^ we transfected circSMAD4 plasmids into NOZ and GBC‐SD cells and found the linear forms detected by primers targeting SMAD4 exon 3 were five‐ to sixfold higher than the control groups, indicating linear forms of circSMAD4 did generate after plasmid transfection (Figure S2A). We then constructed a control plasmid lacking the *Alu* elements necessary for circularization (termed lin‐circSMAD4) (Figure S2B). This approach enabled us to rigorously characterize the biological function of its pure linear products in the cells. As illustrated in Figure S2C, transient transfection resulted in elevated expression of lin‐circSMAD4 in NOZ and GBC‐SD cells. However, lin‐circSMAD4 overexpression failed to enhance cell growth, proliferation, or migratory capacity relative to empty vector controls (Figure S2D–F).

To further validate these findings under stable‐expression conditions, we established NOZ cell lines stably expressing circSMAD4 or lin‐circSMAD4. qRT‐PCR analysis showed that circSMAD4 expression was increased by approximately 25‐fold in the circSMAD4‐stable cells compared with empty vector controls, whereas the corresponding linear transcript was increased by only approximately three‐ to fourfold (Figure S2G). In parallel, lin‐circSMAD4 expression was increased by approximately 27‐fold in the lin‐circSMAD4‐stable NOZ cells relative to empty vector controls (Figure S2H). To determine whether the linear transcript exerted tumour‐promoting activity in vivo, NOZ cells stably expressing lin‐circSMAD4 or empty vector were subcutaneously implanted into nude mice. Tumours harvested on day 28 showed no apparent increase in tumour growth in the lin‐circSMAD4 group (Figure S2I,J). Together, these in vitro and in vivo findings indicate that the linear transcript generated from the circSMAD4 expression construct is unlikely to account for the tumour‐promoting effects observed following circSMAD4 overexpression, supporting a predominant role of the circular RNA itself in GBC progression.

### 3.3 RNA‐binding protein IGF2BP2 induced circSMAD4 expression in gallbladder cancer

Given that circRNA biogenesis is tightly regulated by RBPs that interact with precursor RNAs and the splicing machinery, we sought to identify RBPs potentially responsible for the aberrant upregulation of circSMAD4 in GBC. Because many previously characterized RBPs regulate circRNA formation through binding to flanking intronic regions, our initial analysis focused on candidate RBPs associated with the intronic sequences flanking circSMAD4 (Figure 3A). Nevertheless, RBP‐mediated regulation of circRNA biogenesis is not restricted to flanking introns. For example, ZC3H14 has recently been shown to bind the 5′ and 3′ EIBs of circularized exons and the 3′ UTRs of cognate transcripts to facilitate back‐splicing.^18^ Thus, our intron‐focused analysis was designed to identify candidate regulators of circSMAD4 but does not exclude potential regulation through EIBs or other cis‐regulatory regions. To identify candidate RBPs associated with circSMAD4, we used the ENCORI database (https://rnasysu.com/encori/rbpClipRNA.php?source=circRNA), which catalogues circRNA‐RBP interactions, and the circAtlas v2.0 (https://ngdc.cncb.ac.cn/circatlas/) online database, which provides predicted RBP‐binding motifs across multiple human tissues. From the overlap of these datasets, we obtained 7 RBPs, including U2AF2, ELAVL1, HNRNPC, IGF2BP2, PRPF8, FMR1, and FUS (Figure 3A). We designed two siRNA sequences and an overexpression plasmid for each of these 7 genes and transiently transfected them into NOZ and GBC‐SD cells. We observed that downregulation of all these 7 proteins, except ELAVL1, in GBC‐SD cells led to a significant decrease in the expression level of circSMAD4 in these cells (Figure 3B,C), while only overexpression of HNRNPC and IGF2BP2 could increase the expression level of circSMAD4 (Figure 3D,E). Accordingly, mRNA levels of IGF2BP2 in GBC lines were significantly upregulated compared with L‐2F7 cells (Figure 3F). In addition, we found that IGF2BP2 mRNA expression in 30 paired patient‐derived GBC samples was also greater than that in adjacent non‐cancerous tissues, while HNRNPC levels showed no difference between the two groups (Figure 3G,H). Consistent with elevated circSMAD4 in GBC, IGF2BP2 in GBC samples was positively correlated with circSMAD4 (Figure 3I), whereas IGF2BP2 was not correlated with SMAD4 mRNA (Figure S3A). To further confirm the elevated expression of IGF2BP2 protein in GBC tissues, we performed Western blot analysis on nine pairs of GBC and adjacent tissues, and immunohistochemistry (IHC) analysis on 32 pairs of GBC tissues. Western blot analysis revealed that IGF2BP2 was more highly expressed in GBC tissues compared with adjacent non‐cancerous tissues in 6 of 9 paired samples (66.7%) (Figure 3J). IHC analysis showed that 15 cases out of 32 patients (46.9%) overexpressed IGF2BP2, 12 cases (37.5%) were moderate, and 5 cases (15.6%) were weak, in comparison with minimal levels in all adjacent cholecystitis tissues (Figure 3K,L). We next expanded the clinical cohort to 65 patients to further evaluate IGF2BP2 protein expression and its relationship with circSMAD4. Quantitative analysis of IHC staining demonstrated that IGF2BP2 H‐scores were significantly higher in GBC tissues than in paired adjacent non‐cancerous tissues (Figure 3M). Notably, IGF2BP2 H‐scores in GBC tissues showed a strong positive correlation with circSMAD4 expression across the 65‐patient cohort (*r* = .6408, *p* < .0001; Figure 3N), further supporting a close association between IGF2BP2 abundance and circSMAD4 expression in human GBC.

**FIGURE 3:**
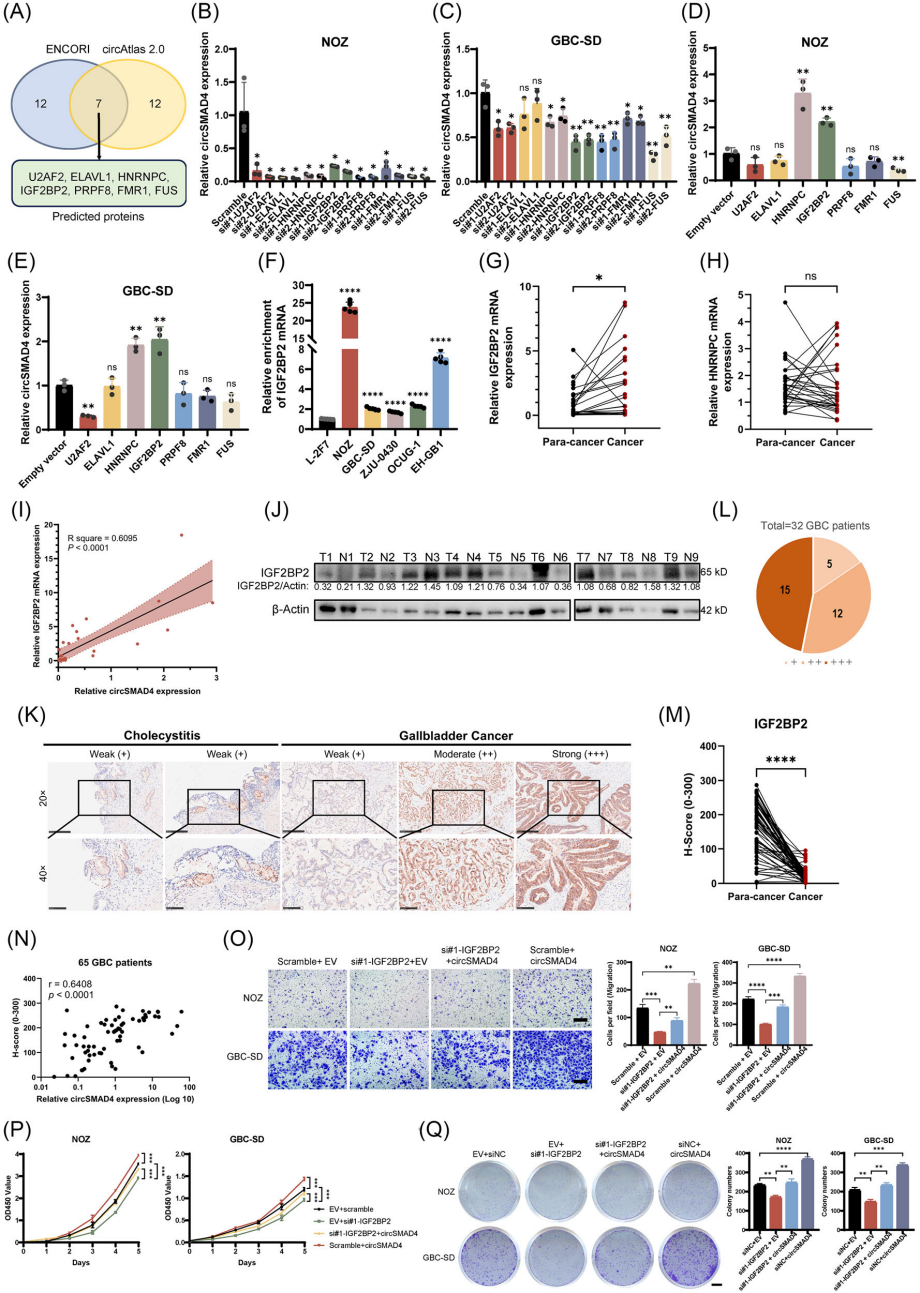
The expression of circSMAD4 is regulated by RNA‐binding protein IGF2BP2. (A) Schematic illustration of predicted upstream RNA‐binding proteins (RBPs) by overlapping ENCORI and circAtlas 2.0 databases. (B, C) circSMAD4 expression levels in (B) NOZ and (C) GBC‐SD cell lines after transfection with knockdown siRNAs of predicted upstream RBPs. Quantitative real‐time PCR analysis; *n* = 3. (D, E) circSMAD4 expression levels in (D) NOZ and (E) GBC‐SD cell lines after transfection with overexpression plasmids of predicted upstream RBPs. Quantitative real‐time PCR analysis; *n* = 3. (F) IGF2BP2 mRNA expression levels in the normal gallbladder epithelial cell L‐2F7 and GBC cell lines, quantitative real‐time PCR analysis; *n* = 3. (G, H) IGF2BP2 and HNRNPC mRNA expression levels in 30 paired GBC cancerous and adjacent non‐cancerous tissues via quantitative real‐time PCR analysis. (I) Spearman correlation analysis between circSMAD4 (*x*‐axis) and IGF2BP2 mRNA (*y*‐axis) expression levels in 30 patient‐derived GBC tissues. (J) IGF2BP2 expression levels in nine pairs of patient‐derived fresh GBC tumour tissues (T) and non‐tumour tissues (N) via western‐blotting analysis. The bands of the Western blot were quantified by ImageJ. (K) Representative images were shown for weak, moderate, and strong expression of IGF2BP2 in IHC staining of patient‐derived cholecystitis and GBC tissues at 20× (top) and 40× (bottom). Scale bars: 250 µm (top), 100 µm (bottom). (L) The distribution of IHC results in 32 GBC tissues. (M) H‐scores of IGF2BP2 expression in 65 paired GBC and adjacent para‐cancer tissues. (N) Spearman correlation analysis between circSMAD4 expression (*x*‐axis) and IGF2BP2 H‐score (*y*‐axis) in 65 patient‐derived GBC tissues. (O) Migration of NOZ and GBC‐SD cells transfected or co‐transfected with IGF2BP2 knockdown siRNA and circSMAD4 plasmids as indicated by transwell assays; *n* = 3. Scale bar: 200 µm. (P, Q) Growth of NOZ and GBC‐SD cells transfected or co‐transfected with IGF2BP2 knockdown siRNA and circSMAD4 plasmids as indicated by (P) CCK‐8 assays; *n* = 3, (Q) colony formation assays. Scale bar: 5 mm; *n* = 3. Data are shown as the mean ± S.D. The *p*‐value was determined by a two‐tailed unpaired Student's *t*‐test. ns, not significant *p < .05, **p < .01, ***p < .001, ****p < .0001.

To further determine whether IGF2BP2 contributes to the maintenance of circSMAD4, we examined circSMAD4 stability following IGF2BP2 depletion. Knockdown of IGF2BP2 markedly accelerated circSMAD4 degradation in NOZ and GBC‐SD cells (Figure S3B,C), indicating that IGF2BP2 not only promotes circSMAD4 expression but also contributes to the stability of mature circSMAD4.

We next investigated whether circSMAD4 functionally mediates the tumour‐promoting effects of IGF2BP2. IGF2BP2 knockdown markedly impaired the migratory capacity of NOZ and GBC‐SD cells, whereas ectopic expression of circSMAD4 substantially rescued this phenotype (Figure 3O). Similarly, the reductions in cell viability and clonogenic growth induced by IGF2BP2 depletion were significantly reversed by circSMAD4 overexpression, as determined by CCK‐8 and colony formation assays, respectively (Figure 3P,Q). Together, these findings identify IGF2BP2 as an upstream regulator of circSMAD4 and support circSMAD4 as an important downstream effector mediating the tumour‐promoting activity of IGF2BP2 in GBC.

### 3.4 The induction of circSMAD4 by IGF2BP2 in GBC depended on m^6^A‐recognition motifs

To elucidate the potential binding region of IGF2BP2 to the flanking sequence of circSMAD4, we constructed 6 FLAG‐tagged deletion mutants for IGF2BP2, including wild type (IGF2BP2‐WT), deletion of KH1 to KH4 domains (△KH1‐4), KH1 and KH2 domains (△KH1‐2), KH3 and KH4 domains (△KH3‐4), KH3 domain (△KH3), and KH4 domain (△KH4) (Figure 4A). The expression of these IGF2BP2 mutants was confirmed by Western blot analysis in GBC‐SD cells (Figure 4B). Following transient transfection with IGF2BP2‐WT and its truncated mutants, we found that only ΔKH1‐4 and ΔKH3‐4 failed to induce an increase in circSMAD4 expression, indicating that KH3‐4 domains are essential to induce circSMAD4 (Figure 4C; Figure S4A).

**FIGURE 4:**
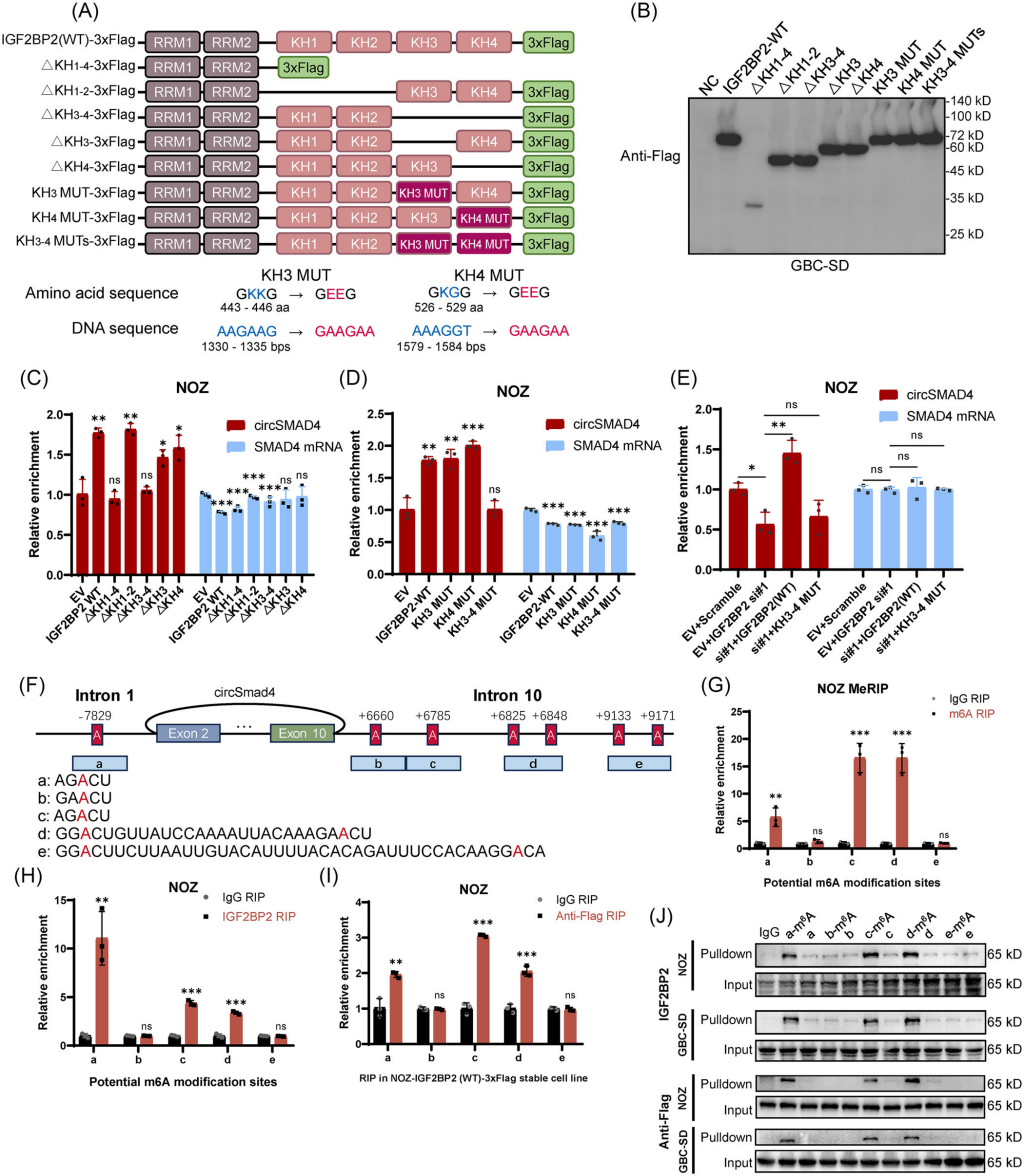
The expression of circSMAD4 is dependent on the m^6^A recognition ability of IGF2BP2. (A) The sketchy map of full‐length FLAG‐tagged wild‐type (WT) IGF2BP2 and the corresponding deletion constructs and point mutants used to map the circSMAD4‐interacting domains. The ‘GxxG’ motifs within the KH3 and KH4 domains, GKKG (amino acids 443‐446) and GKGG (amino acids 526‐529), respectively, were mutated to GEEG (DNA sequence: GAAGAA) to disrupt the canonical RNA‐binding motifs. The corresponding nucleotide substitutions were introduced as indicated. (B) Expression of IGF2BP2 wild‐type (IGF2BP2‐WT) and its various truncations and mutants transfected into GBC‐SD cells was analysed by Western blotting. (C, D) Expression of circSMAD4 and SMAD4 mRNA in NOZ cells transfected with wild‐type IGF2BP2 and its various deletion mutants (C) and point mutants (D) via quantitative real‐time PCR analysis (*n* = 3). (E) circSMAD4 and SMAD4 mRNA relative expression levels in NOZ cells after co‐transfection with the indicated siRNA and mutant plasmids via qRT‐PCR analysis; *n* = 3. (F) The m^6^A modification sites for IGF2BP2 in the flanking sequences of the SMAD4 mRNA transcript were predicted by SRAMP, where each red A was a putative methylation site. (G) m^6^A modification sites in the circSMAD4 pre‐mRNA transcript in NOZ cells via MeRIP assay using anti‐m^6^A antibody; *n* = 3. (H) Putative binding sites of IGF2BP2 in the circSMAD4 pre‐mRNA transcript in NOZ cells via RIP assay using anti‐IGF2BP2 antibody; *n* = 3. (I) Putative binding sites of IGF2BP2 in the circSMAD4 pre‐mRNA transcript in NOZ cells stably expressing IGF2BP2 (WT)‐3xFlag via RIP assay using anti‐Flag antibody. (J) Interaction between IGF2BP2 and predicted m^6^A modification sites in the circSMAD4 pre‐mRNA transcript in parental cells (top) and cells overexpressing IGF2BP2 3xFlag (bottom) via RNA‐protein pulldown assay followed by western blot. a, b, c, d, e for synthesized probes without m^6^A methylation; a‐m^6^A, b‐m^6^A, c‐m^6^A, d‐m^6^A, and e‐m^6^A for synthesized probes with m^6^A methylation. Data are shown as the mean ± S.D. The *p*‐value was determined by a two‐tailed unpaired Student's *t*‐test. ns, not significant *p < .05, **p < .01, ***p < .001, ****p< .0001.

Given the evidence that both HNRNPC and IGF2BP2 are well‐known m^6^A readers.^39^, ^40^ we interrogated whether m^6^A recognition motifs of the IGF2BP2 located within its KH3‐4 domains are critical for the induction of circSMAD4. Three FLAG‐tagged IGF2BP2 point mutants were generated: a KH3 point mutant (KH3 MUT), a KH4 point mutant (KH4 MUT), and double point mutants (KH3‐4 MUTs), in which each motif ‘GxxG’ was uniformly mutated to ‘GEEG’ to presumably disrupt m^6^A‐binding ability^41^ (Figure 4A). Either KH3 MUT or KH4 MUT was capable of enhancing circSMAD4 but not SMAD4 expression as WT; in contrast, the double mutant (KH3‐4 MUTs) failed to elicit the induction (Figure 4D; Figure S4B), indicating that both m^6^A‐binding motifs are required to mediate circSMAD4 induction. To further validate their regulation of circSMAD4, we overexpressed either wild‐type IGF2BP2 (IGF2BP2‐WT) or the KH3‐4 double mutant (KH3‐4 MUTs) in IGF2BP2‐knocked‐down NOZ and GBC‐SD cells (Figure 4E; Figure S4C). While IGF2BP2‐WT effectively restored circSMAD4 expression levels, the KH3‐4 MUTs failed to do so. These findings underscore the regulatory impact of m^6^A‐binding motifs in IGF2BP2 KH3‐4 domains on the expression of circSMAD4.

At the other end, we attempted to define the flanking intronic regions of circSMAD4 associated with IGF2BP2 binding and employed the SRAMP algorithm (http://www.cuilab.cn/m6asiteapp/old) to predict putative m^6^A modification sites in intron 1 and intron 10 proximal to the beginning and end of the circSMAD4 coding area.^42^ The analysis identified one potential high‐confidence m^6^A site in the upstream intronic region and six high‐confidence m^6^A sites in the downstream intron (Figure S4D–G). As demonstrated in Figure 4F, m^6^A modifications at sites in intron 1 designated a, two sites c and d in intron 10, but not at the other two sites b and e, were appreciated to bind to IGF2BP2 in NOZ and GBC‐SD cells in MeRIP assays with an anti‐m^6^A antibody (Figure 4G; Figure S4H). In concert with this observation, RIP assay using anti‐IGF2BP2 showed identical results in which IGF2BP2 bound specifically to sites a, c, and d, but not to sites b and e (Figure 4H; Figure S4I). Furthermore, in NOZ cells stably expressing FLAG‐tagged IGF2BP2, we found similar results in RIP assays using anti‐FLAG antibodies (Figure 4I). As expected, KH3‐4 mutants stably expressed in NOZ and GBC‐SD cells resulted in a complete loss of interaction between the mutants and the predicted m^6^A sites (Figure S4J), supporting the functional importance of the m^6^A recognition motifs. To further investigate the interaction between IGF2BP2 and m^6^A‐methylated regions within circSMAD4 flanking introns, we designed five biotinylated RNA probes corresponding to the seven high‐confidence m^6^A sites. RNA pulldown assays followed by western blot revealed that IGF2BP2 specifically binds to m^6^A‐modified sites a, c, and d, but not to their non‐methylated counterparts or to sites b and e in NOZ and GBC‐SD cells (Figure 4J top). Likewise, in NOZ and GBC‐SD cells stably expressing FLAG‐tagged wild‐type IGF2BP2 yielded the same results (Figure 4J bottom). Collectively, these results demonstrate that the m^6^A recognition capability of the KH3‐4 binding motifs is indispensable for IGF2BP2 binding to specific m^6^A‐modified sites within the flanking intronic regions of circSMAD4, thereby facilitating its expression.

To analyze effects of IGF2BP2 on cell activity, we utilized a series of functional assays in NOZ and GBC‐SD cells. Transwell assays demonstrated that IGF2BP2 knockdown significantly impaired the migratory capacity of GBC cells, whereas overexpression of circSMAD4 effectively rescued this phenotype in IGF2BP2 depletion (Figure 4K). Similarly, the suppression of cell growth and proliferation induced by IGF2BP2 knockdown was reversed by circSMAD4 overexpression in CCK‐8 assays and colony formation assays (Figure 4L,M), supporting the early findings on the structural characterization.

### 3.5 IGF2BP2 regulates the subcellular localization and binding of mature circSMAD4 through its KH3‐4 domains

Having established that IGF2BP2 promotes circSMAD4 biogenesis through m^6^A‐dependent binding to the flanking intronic regions of SMAD4 pre‐mRNA, we next asked whether IGF2BP2 also regulates the subcellular localization of mature circSMAD4 after its formation.

While the alternative splicing and induction of circSMAD4 predominantly occur in the nucleus, circSMAD4 is primarily localized in the cytoplasm of GBC cell lines (Figure 1H). This observation prompted us to investigate the mechanism underlying its subcellular transportation from the nucleus to the cytoplasm, presumably mediated by IGF2BP2. Given the evidence reported previously that an m^6^A reader, YTHDC1, has the ability to mediate the nuclear export of m^6^A‐modified circNSUN2 in colorectal cancer,^43^ we extrapolated that IGF2BP2, another recognized m^6^A reader, likely promotes the nuclear export of circSMAD4.

We began to visualize the subcellular location of circSMAD4 and IGF2BP2 mRNA in the cells via nuclear‐cytoplasmic fractionation assays (Figure 5A,B). The results revealed that circSMAD4 was predominantly localized in the cytoplasm of GBC cell lines, while it was evenly distributed in L‐2F7 cells (Figure 5A). Notably, whereas IGF2BP2 mRNA was primarily retained in the nucleus of L‐2F7 cells, most of the IGF2BP2 transcripts were enriched in the cytoplasm of GBC cell lines (Figure 5B), consistent with the IHC results of IGF2BP2 protein in patient‐derived GBC tissues (Figure 3K). These observations established the subcellular localization pattern and prompted us to investigate whether IGF2BP2 contributes to circSMAD4 nuclear export. We next directly examined whether IGF2BP2 regulates the nuclear‐cytoplasmic distribution of mature circSMAD4. We performed knockdown and overexpression experiments of wild‐type IGF2BP2 in NOZ cells, followed by nuclear‐cytoplasmic fractionation assays. These overexpression and knockdown transduction efficiencies were validated by Western blotting assays (Figure 5C). In addition, the fractionation results revealed a marked increase in cytoplasmic localization of circSMAD4 upon IGF2BP2 overexpression, whereas IGF2BP2 knockdown led to its nuclear retention (Figure 5D), providing strong evidence that IGF2BP2 at least partially promotes the nuclear export of circSMAD4. We next sought to determine whether the nuclear export of circSMAD4 is dependent on the m^6^A‐recognition motifs of IGF2BP2. We then overexpressed either the ΔKH3‐4 truncated mutant or the KH3‐4 MUTs plasmids in the siIGF2BP2 cells. The results revealed that the KH3‐4 MUTs successfully rescued the nuclear retention of circSMAD4 induced by IGF2BP2 knockdown, whereas the ΔKH3‐4 truncation mutant failed to do so (Figure 5E). These findings suggest that the nuclear export of circSMAD4 is dependent on KH3‐4 domains but not the m^6^A‐recognition motifs. To further clarify whether either the KH3, KH4 or both domains are sufficient to shuttle circSMAD4, we overexpressed ΔKH3 and ΔKH4 truncated mutants individually after downregulation of IGF2BP2 in the cells. Both truncated mutants ΔKH3‐4, but not single mutant ΔKH3 or ΔKH4, effectively attenuated the nuclear retention of circSMAD4 induced by IGF2BP2 depletion (Figure 5F), underscoring the functional indispensability of the KH3‐4 domains in mediating circSMAD4 nuclear export. This finding was further validated by IF‐FISH assays in GBC‐SD cells, which yielded consistent results (Figure 5G). Thus, unlike the m^6^A‐dependent role of IGF2BP2 in circSMAD4 biogenesis, its effect on mature circSMAD4 localization appears to depend primarily on the structural integrity of the KH3‐4 domains

**FIGURE 5:**
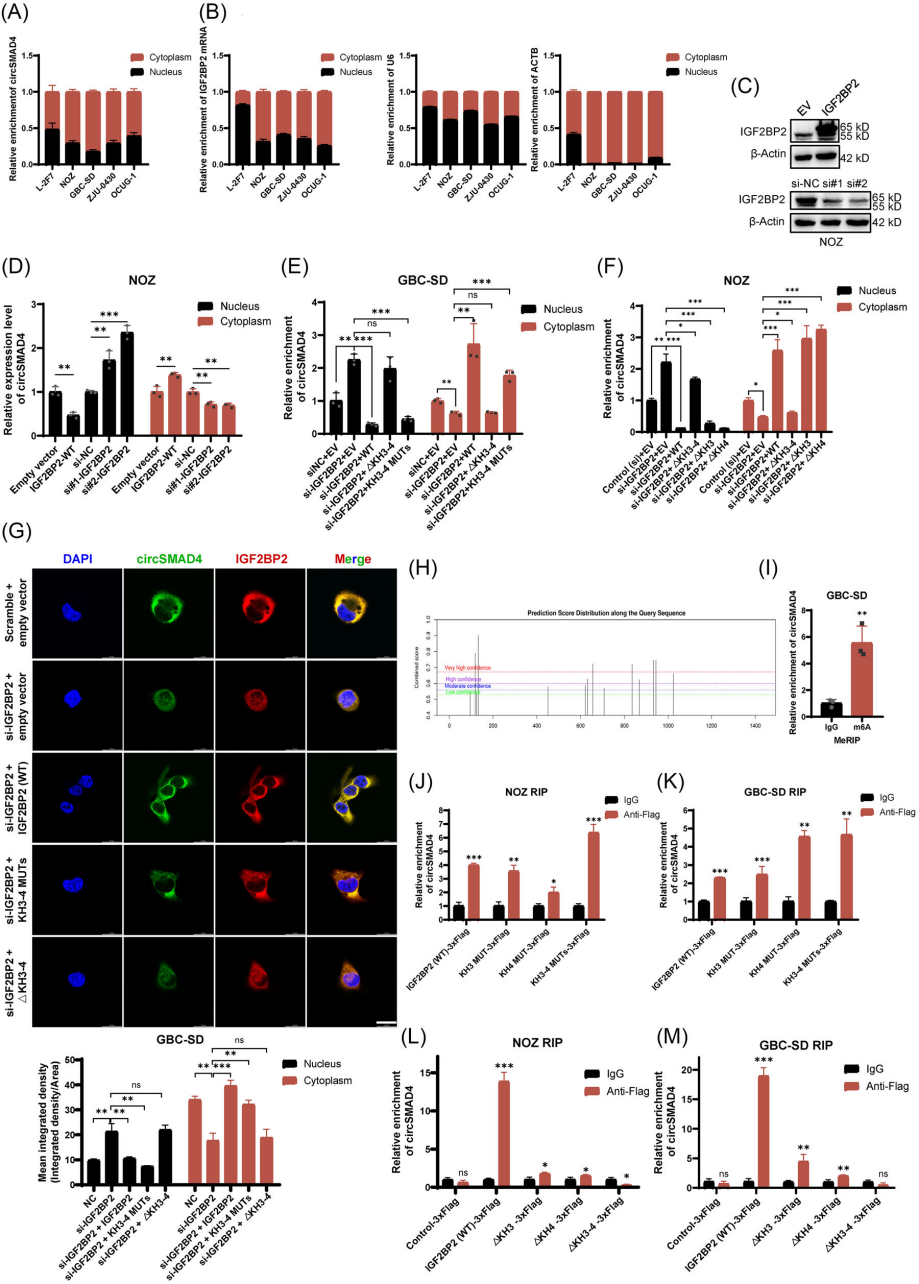
IGF2BP2 promotes circSMAD4 nuclear export independent of m^6^A binding motifs. (A, B) Relative RNA expression levels of circSMAD4 (A) and IGF2BP2 mRNA, U6 and ACTB (B) in the nuclear and cytoplasmic fractions of L‐2F7 and 4 GBC cell lines (NOZ, GBC‐SD, ZJU‐0430, and OCUG‐1) were tested by qRT‐PCR. U6 and ACTB were tested as positive controls for the nuclear‐cytoplasmic fractionation assay; *n* = 3. (C) Expression of IGF2BP2 in NOZ cells transiently transfected with EV, IGF2BP2 overexpression plasmids, si‐NC, si#1‐IGF2BP2 and si2#‐IGF2BP2 via Western blotting assay. EV, empty vector; si‐NC, negative control siRNAs. (D) Nuclear and cytoplasmic circSMAD4 content in the cells expressing wild‐type or siIGF2BP2 via nuclear‐cytoplasmic mRNA fractionation assay followed by qRT‐PCR; *n* = 3. (E) Nuclear and cytoplasmic circSMAD4 content in cells expressing siIGF2BP2 and KH3‐4 deleted or point mutants via a nuclear‐cytoplasmic mRNA fractionation experiment followed by qRT‐PCR; *n* = 3. (F) Nuclear and cytoplasmic circSMAD4 content in cells expressing siIGF2BP2 and single KH3‐ or KH4‐domain deletion using a nuclear‐cytoplasmic mRNA fractionation experiment followed by qRT‐PCR; *n* = 3. (G) Staining of circSMAD4 and IGF2BP2 in the cells expressing siIGF2BP2 and WT, KH3‐4 MUTs, or △KH3‐4 mutant IGF2BP2 via FISH‐IF assay. Scale bar: 20 µm (top); Statistical analysis of mean integrated fluorescence density of circSMAD4 (bottom). (H) m^6^A modification sites in circSMAD4 were predicted using SRAMP. (I) m^6^A modification sites enriched in circSMAD4 via MeRIP assay using anti‐m^6^A antibody; *n* = 3. (J, K) Association of circSMAD4 with IGF2BP2 in (J) NOZ and (K) GBC‐SD cells stably expressing IGF2BP2 various mutants via RIP assay using anti‐Flag antibody; *n* = 3. (L, M) Interaction between circSMAD4 and IGF2BP2 in (L) NOZ and (M) GBC‐SD cells stably expressing IGF2BP2 truncated mutants via RIP assay; *n* = 3. Data are shown as the mean ± S.D. The *p*‐value was determined by a two‐tailed unpaired Student's *t*‐test. ns, not significant *p < .05, **p < .01, ***p < .001.

We next examined if lack of m^6^A methylation of circSMAD4 contributes to its export independent of KH3‐4 m^6^A binding motifs of IGF2BP2. Using the SRAMP algorithm, we predicted potential m^6^A sites across the circSMAD4 sequence, identifying 6 very high, 3 high, and 2 moderate sites (Figure 5H). Subsequently, MeRIP assays using anti‐m^6^A antibodies were performed, confirming a strong enrichment of m^6^A modifications on circSMAD4 (Figure 5I). Thus, these findings support the early notion that circSMAD4 nuclear export partially recapitulates the tumour‐promoting effects mediated by IGF2BP2 and does not rely on classical m^6^A‐recognition mechanisms. Agreed with these results, the point mutation of m^6^A‐binding motifs in KH3 or KH4 did not alter the interaction between circSMAD4 and IGF2BP2 (Figure 5J,K). Notably, deletion of either the KH3 or KH4 domain individually inhibited their interaction; however, concurrent deletion of both domains (ΔKH3‐4 truncation) completely disrupted the binding between IGF2BP2 and circSMAD4 (Figure 5L,M). These findings indicate that either the KH3 or KH4 domain is sufficient to sustain the interaction between IGF2BP2 and circSMAD4; both domains play a dominant role in the nuclear export of circSMAD4.

### 3.6 circSMAD4 enhances IGF2BP2 expression by inhibiting its ubiquitination‐mediated degradation

Previous studies have demonstrated that circRNAs can exert their regulatory functions through various mechanisms, including microRNA sponging, protein translation, and interactions with RNA‐binding proteins.^44^, ^45^, ^46^ To investigate whether circSMAD4 functions via microRNA sponging or protein translation, we first utilized the TransCirc database (https://www.biosino.org/transcirc/) to predict potential open reading frame (ORF) regions within the circSMAD4 sequence. The analysis suggested a putative ORF spanning 1422 nucleotides (Figure S5A). Subsequently, we constructed a plasmid in which a FLAG tag was inserted immediately upstream of the stop codon within the predicted ORF of circSMAD4 and transfected it into NOZ cells together with a FLAG‐tagged SMAD4 mRNA construct as a positive control (Figure S5B). Surprisingly, following transfection, we failed to detect circSMAD4 expression but did find SMAD4 expression in the cells (Figure S5C), suggesting that circSMAD4 is unlikely to be translated into a protein. To further evaluate its translational potential using an independent approach, we performed a ribosome profiling assay and found no obvious binding of circSMAD4 to polysomes (Figure S5D,E), indicating that circSMAD4 is unlikely to undergo efficient translation under the experimental conditions examined. Next, to monitor the possibility of a miRNA sponge that is contingent upon its association with the Argonaute 2 (Ago2) protein, a pivotal component of the RNA‐induced silencing complex (RISC).^47^, ^48^ We measured their association by RIP assays using an anti‐Ago2 antibody. circSMAD4 was not enriched in Ago2‐immunoprecipitated RNA fractions (Figure S5F), excluding the possibility of a canonical miRNA sponge.

Finally, to investigate the potential protein‐binding activity for circSMAD4 in GBC, we performed RNA pulldown assays using a biotinylated antisense probe targeting the back‐splice junction of circSMAD4 and a complementary sense probe as a negative control. The probe pulldown lysates were subjected to silver staining, and a prominent differential band was excised for mass spectrometry analysis (Figure 6A,B). Among the top 15 enriched proteins within the range of 55–70 kDa, IGF2BP2 was identified with notable abundance (Figure 6B). This observation raised the intriguing possibility that IGF2BP2, beyond regulating circSMAD4 expression, might also function as a downstream binding partner mediating its oncogenic role in GBC. To test this possibility with great curiosity, we repeated RNA pulldown assays followed by Western blotting and found that IGF2BP2 was associated with the biotinylated antisense probe, but not with the sense probe control (Figure 6C). Consistently, FISH analysis revealed the predominant co‐localization of the circSMAD4‐IGF2BP2 complex in the cytoplasm of NOZ and GBC‐SD cells (Figure 6D). Neither overexpression nor knockdown of circSMAD4 significantly altered IGF2BP2 mRNA levels in these cells (Figure 6E,F). However, overexpression of circSMAD4 led to a marked increase in IGF2BP2 protein levels (Figure 6G). Conversely, depletion of circSMAD4 using two independent siRNAs consistently reduced IGF2BP2 protein levels in both NOZ and GBC‐SD cells (Figure 6H), suggesting that circSMAD4 regulates IGF2BP2 expression at the translational or posttranslational level.

**FIGURE 6:**
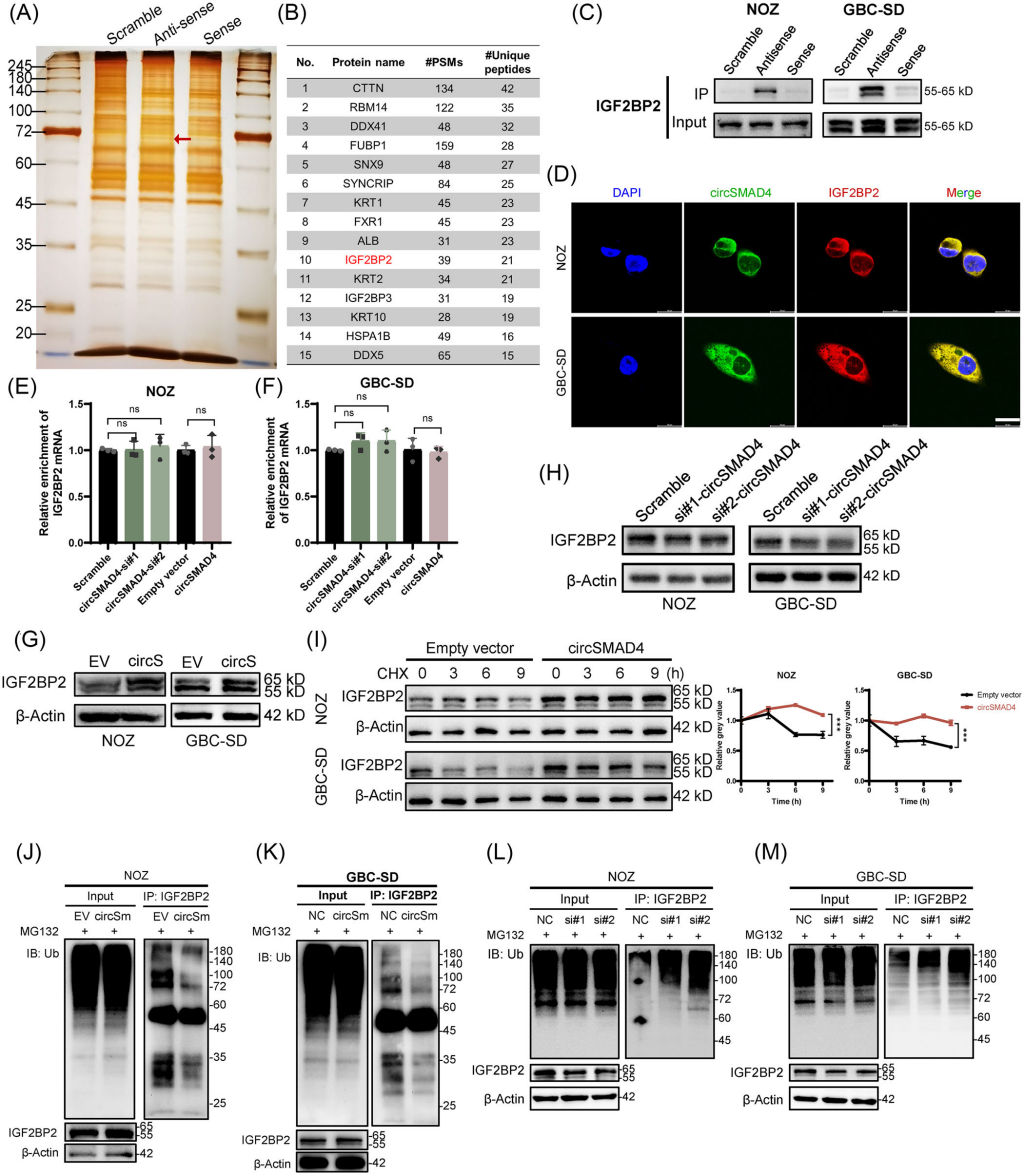
circSMAD4 interacts with IGF2BP2, and both stabilize each other to enhance their expression. (A) Silver staining of cell lysate samples pulled down with biotinylated anti‐sense or sense probes of circSMAD4. A red arrow shows an interesting band subjected to mass spectrometry analysis. (B) List of the top 15 circSMAD4‐related proteins. PSMs: Peptide spectrum matches. (C) Association between circSMAD4 and IGF2BP2 in cells pulled down with biotinylated RNA probes followed by WB. (D) The subcellular co‐localization of IGF2BP2 and circSMAD4 in NOZ and GBC‐SD cells in IF and FISH assays. Nuclei were stained with DAPI. Scale bar: 20 µm. (E, F) Expression level of IGF2BP2 mRNA in (E) NOZ and (F) GBC‐SD cells expressing circSMAD4 knockdown or overexpression in qRT‐PCR assay. (G‐H) Expression of IGF2BP2 in cells with transient circSMAD4 overexpression (G) or circSMAD4 knockdown (H) via Western blot assay. EV, empty vector; circS, circSMAD4. (I) IGF2BP2 stability in NOZ and GBC‐SD cells transfected with circSMAD4 plasmids in the presence of 20 µM CHX for the indicated time. Protein extracts were subjected to an immunoblotting assay (top). Quantification curve of the relative expression levels of IGF2BP2 protein (bottom); *n* = 3. (J–M) Effects of circSMAD4 on IGF2BP2 ubiquitination in NOZ and GBC‐SD cells. For circSMAD4 overexpression experiments, NOZ (J) and GBC‐SD (K) cells were transfected with empty vector (EV) or circSMAD4 (circS) plasmids for 48 h, followed by treatment with 20 µM MG132 for 6 h. For circSMAD4 knockdown experiments, NOZ (L) and GBC‐SD (M) cells were transfected with scramble control, si#1‐circSMAD4 (si#1), or si#2‐circSMAD4 (si#2), followed by MG132 treatment under the same conditions. IGF2BP2 was immunoprecipitated using an anti‐IGF2BP2 antibody, and its ubiquitination was assessed by immunoblotting (IB) with an anti‐ubiquitin antibody. Data are shown as the mean ± S.D. The *p*‐value was determined by a two‐tailed unpaired Student's *t*‐test. ns, not significant; ***p < .001.

To test if circSMAD4 regulates IGF2BP2 protein stability, we treated NOZ and GBC‐SD cells with cycloheximide (CHX), a translation inhibitor that blocks de novo protein synthesis. circSMAD4 overexpression attenuated IGF2BP2 protein degradation (Figure 6I), suggesting that circSMAD4 stabilizes IGF2BP2. Previous studies have reported that certain long non‐coding RNAs (lncRNAs) can stabilize IGF2BP2 by suppressing its ubiquitination.^49^ To assess the ubiquitination status of IGF2BP2 in GBC cells, we performed co‐immunoprecipitation (co‐IP) assays and found a marked reduction in IGF2BP2 polyubiquitination levels in cells overexpressing circSMAD4 relative to control vector cells (Figure 6J,K). Conversely, depletion of circSMAD4 markedly increased IGF2BP2 polyubiquitination in both cell lines (Figure 6L,M). Together, these complementary gain‐ and loss‐of‐function findings demonstrate that circSMAD4 stabilizes IGF2BP2 protein by suppressing its ubiquitination‐mediated degradation.

### 3.7 circSMAD4 drives gallbladder cancer by activating the IGF2BP2/JAK2/STAT3 signalling axis

To dissect downstream molecular mechanisms driven by the circSMAD4‐IGF2BP2 axis in gallbladder cancer, we conducted bulk RNA‐sequencing on GBC‐SD cells stably overexpressing circSMAD4 or carrying empty vectors. Differential‐expression analysis uncovered 60 most significantly altered genes in the heat map (Figure 7A). Gene‐set enrichment analysis (GSEA) further showed that the IL‐6/JAK/STAT3 signalling cascade and the epithelial–mesenchymal transition (EMT) hallmark pathway were highly upregulated in circSMAD4‐expressing transcripts (Figure 7B). Subsequently, a number of key regulators involved in both signalling pathways were measured via western blot. circSMAD4 overexpression markedly augmented the mesenchymal markers N‐cadherin and MMP‐9 and the phosphorylated forms of JAK2 and STAT3, while concomitantly attenuating the epithelial marker E‐cadherin, whereas total JAK2 and STAT3 levels remained unaltered (Figure 7C). Collectively, these data confirm that circSMAD4 robustly activates both EMT and the JAK/STAT3 signalling cascades in gallbladder cancer cells.

**FIGURE 7:**
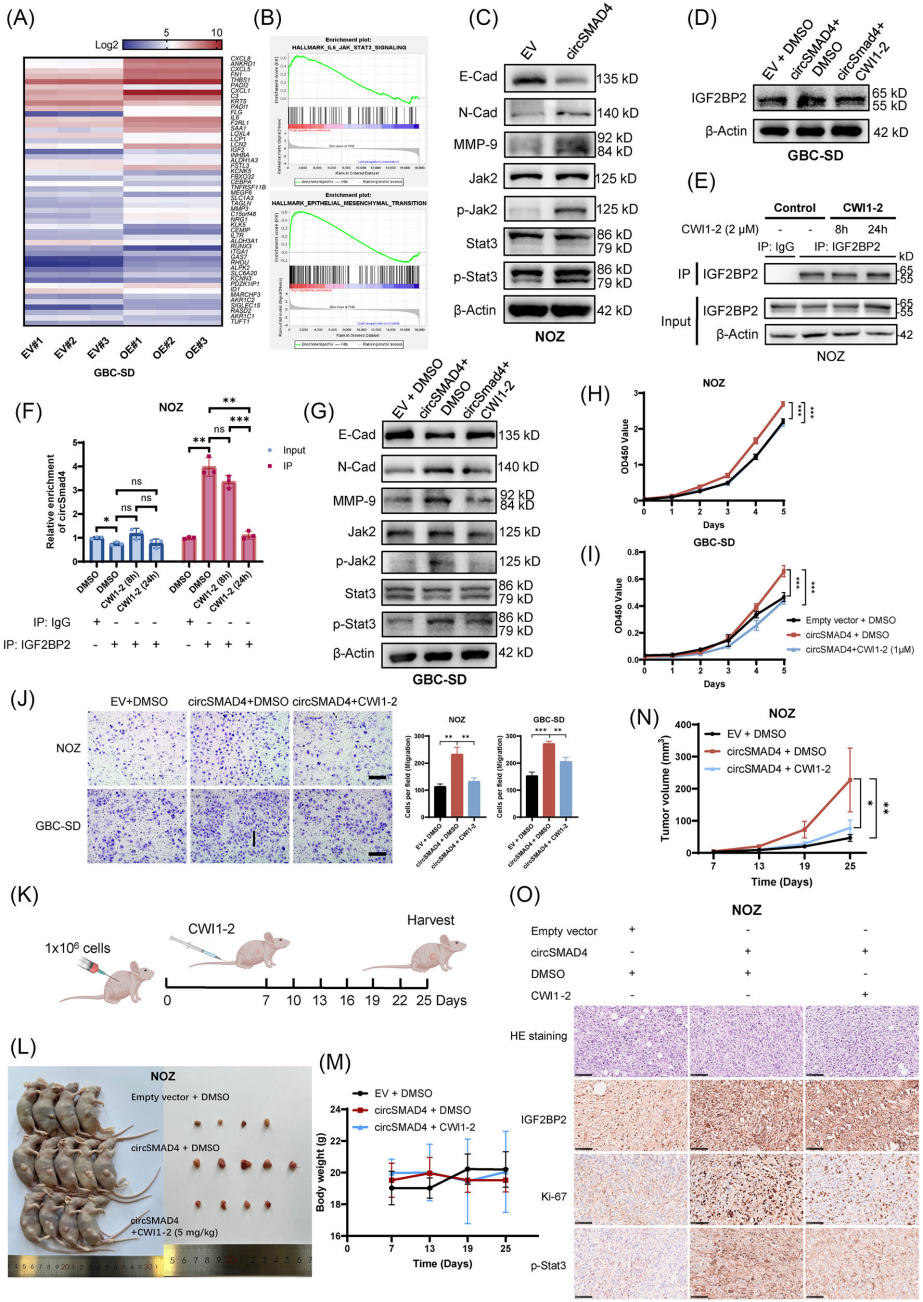
circSMAD4 promotes GBC development through the IGF2BP2/JAK2/STAT3 and EMT pathways. (A) Heatmap showing the RNA‐seq profiles in empty vector and circSMAD4‐overexpressing GBC‐SD cells. (B) Gene set enrichment analysis (GSEA) showing EMT and JAK/STAT pathways from the empty vector (EV) and circSMAD4 overexpression group (circSMAD4). (C) Expression levels of EMT‐ and JAK2/STAT3 related proteins in NOZ cells with empty vector (EV) and circSMAD4 overexpression by WB. (D) IGF2BP2 protein levels in NOZ cells transfected with circSMAD4 and treated with 2 µM CWI1‐2 by WB. (E, F) Effects of CWI1‐2 (2 µM) on the interaction between IGF2BP2 and circSMAD4 in NOZ cells. Cells were treated with DMSO or CWI1‐2 for 8 or 24 h, followed by immunoprecipitation with anti‐IgG or anti‐IGF2BP2 antibodies. (E) IGF2BP2 protein levels in the input lysates and immunoprecipitated fractions were examined by immunoblotting. (F) circSMAD4 enrichment in IGF2BP2 immunoprecipitates was quantified by RIP‐qPCR to assess the interaction between IGF2BP2 and circSMAD4; *n* = 3. (G) Expression levels of EMT and JAK2/STAT3 related proteins in GBC‐SD cells with empty vector (EV), circSMAD4 overexpression and CWI1‐2 (2 µM) treatment after circSMAD4 overexpression in WB. (H–J) Effects of CWI1‐2 on circSMAD4‐induced proliferation and migration in NOZ and GBC‐SD cells. Cells overexpressing circSMAD4 were treated with DMSO or 2 µM CWI1‐2, and cell proliferation was assessed by CCK‐8 assays (H, I), while migratory capacity was evaluated by Transwell assays (J). Representative Transwell images are shown. Scale bar: 200 µm; *n* = 3. (K) Treatment scheme of xenograft models treated with DMSO or 5 mg/kg CWI1‐2. 1 × 10^6^ NOZ or GBC‐SD cells were injected subcutaneously in nude mice. Treatment with CWI1‐2 was initiated on post‐injection day 7 and continued every 3 days. Xenograft tumours were harvested on post‐injection day 25. (Created in https://BioRender.com) (L) The above models with NOZ cells were imaged on day 25. (M, N) Changes in mouse body weight (M) and tumour growth over time (N). (O) Representative images showing HE staining and IHC for expression levels of IGF2BP2, Ki67, and p‐STAT3 proteins in tumours. Scale bar: 100 µm.

Finally, in an attempt to seek the therapeutic potential in the treatment of GBC, we particularly focused on CWI1‐2, a small molecule compound capable of disrupting IGF2BP2 binding to m^6^A‐modified RNAs.^40^ Treatment of GBC‐SD cells stably overexpressing circSMAD4 with CWI1‐2 did not significantly affect expression levels of IGF2BP2 to the control that was induced by overexpression of circSMAD4 (Figure 7D). To further determine whether CWI1‐2 interferes with the interaction between IGF2BP2 and circSMAD4, we performed IGF2BP2 immunoprecipitation followed by RIP‐qPCR analysis. Comparable IGF2BP2 protein levels were observed in the input lysates across all treatment groups, and similar amounts of IGF2BP2 were immunoprecipitated following DMSO treatment or CWI1‐2 exposure for 8 or 24 h (Figure 7E). Notably, CWI1‐2 treatment for 8 h did not significantly alter circSMAD4 enrichment in IGF2BP2 immunoprecipitates, whereas treatment for 24 h significantly reduced the association between circSMAD4 and IGF2BP2 (Figure 7F). These findings indicate that prolonged CWI1‐2 exposure disrupts the circSMAD4‐IGF2BP2 interaction without reducing IGF2BP2 protein abundance.

Having confirmed disruption of the circSMAD4‐IGF2BP2 interaction after CWI1‐2 treatment, we next examined its downstream consequences. CWI1‐2 markedly increased E‐cadherin while attenuating N‐cadherin, MMP‐9, and the phosphorylated forms of JAK2 and STAT3 (Figure 7G), blocking the EMT program and the JAK2/STAT3 signalling cascade induced by circSMAD4. Accordingly, the functional analyses showed that CWI1‐2 inhibited proliferation and migration in NOZ and GBC‐SD cells induced by circSMAD4 (Figure 7H–J; Figure S6A). These findings demonstrate that CWI1‐2 acts as an effective blocker of the interaction between IGF2BP2 and circSMAD4 in gallbladder cancer cells. To evaluate its efficacy in vivo, we treated CWI1‐2‐treated tumour models of NOZ or GBC‐SD cells stably expressing either an empty vector or circSMAD4 in BALB/c nude mice. After a course of 7 treatments with CWI1‐2 every 3 days, tumours were removed (Figure 7K). CWI1‐2 markedly attenuated the circSMAD4‐driven proliferative advantage, resulting in significantly smaller tumour volumes without appreciably affecting mouse body weight (Figure 7L–N; Figure S6B,C). Accordingly, H&E staining and immunohistochemistry demonstrated circSMAD4‐induced upregulation of IGF2BP2, Ki‐67, and phosphorylated STAT3, all of which were restored to near‐baseline levels upon CWI1‐2 treatment (Figure 7O; Figure S6D). Collectively, the transcriptomic, biochemical, and functional data suggest a mechanistic axis whereby circSMAD4 drives gallbladder cancer progression through its partnership with IGF2BP2. These findings provide preclinical evidence that pharmacological disruption of the circSMAD4–IGF2BP2 interaction may represent a potential strategy for attenuating circSMAD4‐dependent tumour growth.

## 4 DISCUSSION

Over the past decade, a large volume of research has focused on the biological and pathologic function of circular RNAs in a variety of human diseases, including cancers.^50^ However, the tumorigenic activity of circular RNAs through protein‐binding mechanisms in tumour progression is poorly understood. In this study, we characterized hsa_circ_0108590 (circSMAD4), a SMAD4‐derived circRNA located at chromosome 18q21.2, as an oncogenic regulator of GBC progression. Interestingly, we unveiled that the biogenesis and circularization of circSMAD4 rely on the binding activity of an RNA‐binding protein, IGF2BP2, in an m^6^A‐dependent manner. Specifically, the GxxG motifs within the KH3 and KH4 domains of IGF2BP2 are required for m^6^A recognition, as mutation or deletion of these domains markedly impaired circSMAD4 expression. Consistent with these findings, the binding ability of IGF2BP2 to circSMAD4 in the cytoplasm resulted in the stabilization of circSMAD4 levels. Thus, our data demonstrate that IGF2BP2 is essential for the regulation of circSMAD4 expression and function in GBC. On the other hand, the flanking intronic sequences (a, c, d) harbouring the G/AACU motif of pre‐mRNA SMAD4 determined its association with IGF2BP2, thereby regulating circSMAD4 splicing and circularization. This finding was in line with previous reports that upstream and downstream flanking sequences of circRNAs contributed to their circulation and biogenesis.^51^ Nevertheless, RBP‐mediated regulation of circRNA biogenesis is not necessarily restricted to flanking intronic regions, and additional cis‐regulatory elements, including EIBs, may also contribute to circSMAD4 formation. Although the precise IGF2BP2‐binding sequences within mature cytoplasmic circSMAD4 remain to be defined, our data support an m^6^A‐dependent interaction between circSMAD4 and IGF2BP2. However, the specific m^6^A‐modified nucleotide responsible for circSMAD4 biogenesis and IGF2BP2 recognition has not yet been resolved. Definitive identification of these functional sites will require single‐nucleotide‐resolution m^6^A mapping combined with site‐directed mutagenesis in future studies.

Given that circSMAD4 originates from the SMAD4 locus, its potential crosstalk with canonical TGF‐β/SMAD signalling is an obvious question. Interestingly, we found that circSMAD4 is significantly upregulated in GBC while linear SMAD4 mRNA levels remain unchanged, indicating that the circular transcript is regulated independently of its host gene. However, our current study did not directly test whether circSMAD4 alters the canonical TGF‐β cascade, such as SMAD2/3 phosphorylation or nuclear translocation. Thus, we cannot rule out the possibility that classical TGF‐β signalling contributes to the phenotypes we observed, which remains an important direction for future research.

Of note, circSMAD4 overexpression reduced, whereas circSMAD4 depletion increased, IGF2BP2 polyubiquitination. Together with the reciprocal changes in IGF2BP2 protein abundance following circSMAD4 overexpression or depletion, these findings suggest that circSMAD4 stabilizes IGF2BP2, at least in part, by suppressing its ubiquitination‐associated degradation. However, the specific E3 ubiquitin ligase responsible for IGF2BP2 ubiquitination and degradation in GBC remains unknown. Identifying the relevant E3 ligase and determining whether circSMAD4 interferes with E3‐ligase recruitment, substrate recognition, or ubiquitin transfer to IGF2BP2 will be important directions for future investigation. In addition to ubiquitination, IGF2BP2 may also be regulated by other post‐translational modifications, such as phosphorylation, which could influence its protein stability, subcellular localization, RNA‐binding activity, or interactions with partner proteins. Potential crosstalk between these post‐translational modifications (PTMs) and ubiquitination may therefore provide an additional layer of regulation of IGF2BP2 function and warrants further investigation. Because a direct proteasome‐rescue experiment was not performed, the precise contribution of proteasomal degradation to circSMAD4‐mediated IGF2BP2 stabilization also remains to be further defined. These reciprocal regulatory effects between circSMAD4 and IGF2BP2 may establish a positive feedback loop that cooperatively promotes GBC progression.

In addition, elevated circSMAD4 and IGF2BP2 expression in GBC was associated with more aggressive disease and poorer prognosis. However, multivariable Cox regression analysis was not performed in the present study. Therefore, whether circSMAD4 serves as an independent prognostic factor beyond established clinicopathological variables remains to be determined. Further validation in larger, well‐characterized clinical cohorts with multivariable survival analyses is warranted.

To further place circSMAD4 expression in a broader biological context, we examined its distribution across normal human tissues using the circBank database. circSMAD4 exhibited a heterogeneous and relatively restricted tissue expression pattern, with the highest expression observed in the brain and lower expression in the cerebellum, whereas its expression was low or nearly undetectable in most other tissues examined, including liver, lung, kidney, gastrointestinal tissues, skeletal muscle, skin, and reproductive tissues. These observations suggest that circSMAD4 is not ubiquitously expressed across normal human tissues. Nevertheless, because circBank primarily provides tissue‐level expression data rather than systematic tumour‐versus‐normal comparisons across cancer types, these findings do not establish that circSMAD4 is specific to GBC. Further pan‐cancer analyses will therefore be required to determine whether circSMAD4 upregulation represents a GBC‐enriched event or a broader cancer‐associated phenomenon. Taken together, to our knowledge, this study provides the first mechanistic evidence that hsa_circ_0108590 cooperates with IGF2BP2 to promote GBC progression, highlighting the circSMAD4/IGF2BP2 axis as a potential therapeutic target in GBC.

Although some circRNAs have been reported to encode functional peptides through cap‐independent translation mechanisms, our current data do not support efficient translation of circSMAD4 under the experimental conditions examined. A predicted ORF was identified within circSMAD4, but no detectable circSMAD4‐derived FLAG‐tagged product was observed following expression of the corresponding construct. Consistently, circSMAD4 showed no obvious association with polysome fractions, further arguing against active translation. However, these negative findings should not be interpreted as definitive exclusion of coding potential, as low‐abundance or context‐dependent translation, including IRES‐ or m^6^A‐driven translation, may not be captured under the conditions tested.^52^, ^53^ Additional studies using dedicated translation reporters or high‐resolution ribosome profiling would be required to fully exclude such possibilities. Likewise, the absence of circSMAD4 enrichment in Ago2 immunoprecipitates argues against a major role as a canonical Ago2‐dependent miRNA sponge. Together, these findings support a predominant protein‐interaction‐based mechanism for circSMAD4 in GBC while acknowledging that alternative functions cannot be completely excluded.

CircRNAs are initially generated and retained in the nucleus following alternative splicing before they mainly function in the cytoplasm, suggesting that their export to the cytoplasm is an essential mechanism necessary for exerting downstream functions. The mechanisms governing the nuclear export of circRNAs remained largely enigmatic and underexplored. Recent studies have shown that YTHDC1^43^ and IGF2BP1,^54^ both of which are m^6^A readers, can export circRNAs in an m^6^A‐dependent manner. In addition, the IGF2BP family plays a pivotal role in post‐transcriptional regulation of mRNAs and lncRNAs, and is known to form granule‐like mRNA structures in the perinuclear region.^55^ In our study, we identified the predominant location of circSMAD4 in the cytosol and a partial role played by IGF2BP2 in circSMAD4 nuclear exportation. IGF2BP2, another m^6^A‐recognition binding protein, partially facilitated circSMAD4 nuclear exportation, whereas point mutation of KH3 and KH4 m^6^A‐binding motifs failed to recapitulate this activity. These studies implicate that other domains of KH3 and KH4 contribute in part to circSMAD4 shuttling from nucleus to cytoplasm. This property of IGF2BP2 was distinct from YTHDC1, which requires m^6^A modifications for circRNA nuclear export.^43^ Given that IGF2BP2 is capable of forming heterodimers with its paralogs IGF2BP1 and IGF2BP3^56^ to commit pathologic activity, it therefore needs substantial efforts to decipher novel RNA‐binding proteins and partners that primarily account for the nuclear‐cytosol transportation of circSMAD4. It is also of interest that, although IGF2BP2 was predominantly localized in the cytoplasm, a detectable fraction was present in the nucleus. Our data support a functional role for this nuclear IGF2BP2 fraction in regulating circSMAD4 biogenesis and expression. However, because IGF2BP2 lacks a canonical nuclear localization signal (NLS),^57^ the molecular mechanism responsible for its nuclear entry remains unclear. Potential mechanisms may involve passive diffusion, piggyback transport through interaction with other nuclear proteins, or association with nuclear transport factors, but these possibilities were not directly examined in the present study. Defining the machinery responsible for IGF2BP2 nuclear translocation will require dedicated biochemical and protein‐interaction studies and represent an important direction for future investigations. Accordingly, our current conclusions are limited to the functional involvement of nuclear IGF2BP2 in circSMAD4 biogenesis rather than the mechanism by which IGF2BP2 is transported into the nucleus.

In the search for circSMAD4‐binding proteins with mass spectrometry on silver staining candidates, we were particularly interested in IGF2BP2, which was listed among the top 10 ranked candidates, based on the early findings with nuclear function regulating circSMAD4 expression. In fact, the other top candidates such as CTTN and RBM14 remain to be further characterized, with curiosity about their interaction with circSMAD4. For example, RBM subfamily members RBMS2 and RBMS3 stabilized P21 mRNA and LIMS1 to contribute to the development of breast cancer and colon cancer, respectively.^58^, ^59^ Notably, the reduction in IGF2BP2 observed in CWI1‐2‐treated xenograft tumours differed from the unchanged IGF2BP2 abundance observed following short‐term CWI1‐2 treatment in vitro. Given the substantially longer and more complex in vivo treatment context, this reduction may reflect cumulative disruption of the circSMAD4‐IGF2BP2 regulatory axis and secondary consequences of sustained pathway inhibition and altered tumour biology, rather than a direct effect of CWI1‐2 on IGF2BP2 degradation. Nevertheless, our preclinical findings with CWI1‐2 suggest that pharmacological disruption of the circSMAD4‐IGF2BP2 interaction may represent a potential therapeutic strategy for GBC, although further validation in clinically relevant models, together with additional pharmacological and safety assessments, will be required before clinical translation. In conclusion, we provide the first comprehensive evidence that m^6^A‐dependent IGF2BP2‐induced circSMAD4 functions as a key oncogenic circRNA and a potential diagnostic/prognostic biomarker in GBC. circSMAD4 contributes to GBC progression in cooperation with IGF2BP2, with formation and mutual stabilization of the circSMAD4/IGF2BP2 complex accompanied by JAK2/STAT3‐ and EMT‐related molecular changes. This process and collaboration model rely specifically on m^6^A modification of the KH3‐4 domains of IGF2BP2 (Graphical abstract).

Notably, our study reveals that IGF2BP2, a previously unrecognized dual‐function RBP in circRNA biology, acts both as an upstream regulator of circRNA biogenesis and a downstream effector. This bifunctional role underscores the context‐dependent versatility of IGF2BP2 in cancer. Moreover, our findings help understand circRNA subcellular dynamics and highlight the circSMAD4/IGF2BP2 complex as a potential therapeutic target in GBC, especially in tumours with IGF2BP2 hyperactivity.

## 5 CONCLUSION

Our study characterizes hsa_circ_0108590 (circSMAD4) as an oncogenic SMAD4‐derived circRNA in gallbladder cancer. Mechanistically, IGF2BP2 promotes circSMAD4 biogenesis and contributes to its stabilization in an m^6^A‐dependent manner, whereas circSMAD4 reciprocally stabilizes IGF2BP2, at least in part, by suppressing its ubiquitination‐associated degradation. This reciprocal regulation supports a positive feedback loop that promotes GBC progression. Transcriptomic enrichment and selected protein‐level analyses further indicate that this regulatory axis is associated with enhanced JAK2/STAT3 signalling and EMT‐related molecular changes. Moreover, CWI1‐2 disrupted the circSMAD4‐IGF2BP2 interaction and suppressed tumour growth in xenograft models, providing preclinical proof‐of‐concept for pharmacological targeting of this regulatory axis. Collectively, our findings reveal a previously uncharacterized circSMAD4‐IGF2BP2 regulatory mechanism in GBC and highlight m^6^A‐dependent circRNA‐RBP interactions as a potential therapeutic vulnerability.

## AUTHOR CONTRIBUTIONS

Y.L., Y.X., and Y.Z. contributed equally to this work. Y.L., Y.X., H.X., and M.L. designed the research. Y.L., Y.X., Y.Z., Z.Y., L.G., S.G., Z.W., and S.Q. performed experiments and analyzed the data. Y.X. and Y.L. collected human GBC samples. Y.L. and Y.X. wrote the manuscript. Y.L., R.S., and Y.B.L. revised the manuscript.

## CONFLICT OF INTEREST STATEMENT

The authors declare no conflict of interest.

## ETHICS STATEMENT

This study was approved by the Ethics Committee of Renji Hospital, Shanghai Jiao Tong University School of Medicine (Approval No. KY2024‐036‐C), and conducted in accordance with the ethical principles outlined in the Declaration of Helsinki.

## CONSENT FOR PUBLICATION

All authors have read and approved the final manuscript.

## Supporting information



Supporting Information

## Data Availability

The circRNA sequencing raw data generated in this study have been deposited in the National Genomics Data Center (NGDC) under project accession PRJCA064392. The mass spectrometry raw data have been deposited in the iProX repository under Project ID: IPX0020111000. All other data supporting the findings of this study are available from the corresponding author upon reasonable request.
