# Supplementary material for: Circular RNA SMAD4 coordinates with m6A‐dependent IGF2BP2 to promote gallbladder cancer development
Source: Clin Transl Med. 2026 Nov 5;16(11):e70892. doi: 10.1002/ctm2.70892 (PMC13735908; doi:10.1002/ctm2.70892)
Supplement: Supplementary file 1 [file CTM2-16-e70892-s001.docx]

**Circular RNA SMAD4 Coordinates with m^6^A-Dependent IGF2BP2 to Promote Gallbladder Cancer Development**

Yiming Li^1,2,3,^**^※^**, Yayun Xu^1,2,3,^**^※^**, Yuan Zhang^1,2,3,^**^※^**, Si Gao^1,2,3^, Lili Gao^4^, Zhiqing Yuan^1,2,3^, Ziyi Wang^1,2,3^, Shimei Qiu^2^, Peipei Xu^1,2,3^, Yuanding Liu^1,2,3^, Zhaonan Liu^1,2,3^, Huijie Miao^1,2,3^, Hang Xu^2,3^, Maolan Li^1,2^, Rong Shao^2,3,5,^*, Yingbin Liu^1,2,3,^*

**Contents**

Materials and methods

References

Supplementary figures and figure legends

**Materials and methods**

*Clinical specimens*

Human gallbladder cancer (GBC) tissues and paired adjacent non-tumorous specimens were obtained from 65 patients who underwent surgical resection at Renji HospitalAffiliated to Shanghai Jiao Tong University School of Medicine from 2020 to 2024. The diagnosis of GBC was confirmed independently by experienced pathologists at Renji Hospital. Detailed clinicopathological characteristics of the enrolled patients are provided in Supplementary Table 3. Tumor grading and clinical staging were determined based on the criteria of the 8th edition of the American Joint Committee on Cancer (AJCC). All patients were subjected to regular postoperative follow-up for clinical outcome monitoring and survival analysis.

*Cell lines and cell culture*

Human gallbladder cancer (hGBC) cell lines NOZ (RRID:CVCL_3079), GBC-SD (RRID:CVCL_6903), ZJU-0430 (RRID:CVCL_XE03), OCUG-1 (RRID:CVCL_3083), EHGB-1 and HEK-293T (RRID:CVCL_0063) were purchased from Mingzhou Biotechnology (Ningbo, Zhejiang Province, China) and Shanghai FuHeng Biotechnology (Shanghai, China). Human normal gallbladder epithelial cell line L-2F7 (RRID:CVCL_C9FM, PMID: 36387215) was established in our lab^1^. All cell lines were contamination free and were authenticated by STR (Short tandem repeat). All hGBC cell lines were cultured in DMEM high glucose medium (Cat. 41401ES76, Yeasen, Shanghai, China) with 10% fetal bovine serum (FBS) (Cat. 164210, Procell, Wuhan, Hubei Province, China) and 1% penicillin-streptomycin solution (Cat. 60162ES76, Yeasen, Shanghai, China). L-2F7 cells were cultured in the Epithelial Cell Medium (EpiCM) (Cat. 4101, ScienCell, California, USA) with 10% FBS (Cat. 164210, Procell, Hubei Province, China) and 1% penicillin-streptomycin solution (Cat. 60162ES76, Yeasen, Shanghai, China). All adherent cells were digested with trypsin solution (Cat. 40127ES60, Yeasen, Shanghai, China) for 3 min before cell passage or being frozen by using serum-free cell saving buffer (Cat. C30100, NCM Biotech, Jiangsu Province, China).

*Cell and tissue RNA isolation and qRT-PCR*

Cellular total RNA was isolated according to the manufacturer guide of RNA Isolation Kit (Cat. B0004D, EZBioscience, Roseville, USA), and tissue total RNA was isolated with Trizol reagent. RNA concentrations were quantified by the NanoDrop 2000c spectrophotometer (Thermo scientific, Massachusetts, USA), RNA was treated with gDNA wiper and then reverse transcribed to cDNA according to manufacturer’s manual of HiScript® II Q Select RT SuperMix(Cat. R233-01, Vazyme, Nanjing, Jiangsu Province, China). Quantitative PCR reaction mix was prepared according to the guidelines of ChamQ Universal SYBR qPCR Master Mix (Cat. Q711-02/03, Vazyme, Nanjing, Jiangsu Province, China) and run in a qRT-PCR system (QuantStudio Dx, Applied Biosystem, Massachusetts, USA). All the primers used in this study are listed in Supplementary Table 1.

*Co-immunoprecipitation and ubiquitination assay*

Cells subjected to the indicated treatments were washed twice with ice-cold PBS and lysed on ice in Western/IP lysis buffer (Beyotime, #P0013) supplemented with protease inhibitor cocktail (Beyotime, #ST506). Cell lysates were clarified by centrifugation at 14,000×g for 10 min at 4°C, and protein concentrations were determined using a BCA protein assay kit (Beyotime, #P0010). Equal amounts of total protein were incubated with anti-IGF2BP2 antibody or the corresponding normal IgG control overnight at 4°C with gentle rotation. Protein A/G beads (Selleckchem, #B23202) were then added and incubated for an additional 6 h at 4°C. The immune complexes were collected and washed three times with ice-cold Western/IP lysis buffer. Bound proteins were eluted by boiling the beads in SDS sample buffer and subsequently analyzed by Western blotting.

For ubiquitination assays, cells were treated with MG132 at 2 μM for 6 h before harvest to inhibit proteasomal degradation and facilitate the accumulation of ubiquitinated proteins. IGF2BP2 was then immunoprecipitated as described above, and the precipitated proteins were subjected to immunoblotting with an anti-ubiquitin antibody (CST, #3933) to determine IGF2BP2 polyubiquitination levels. IGF2BP2 immunoblotting of the immunoprecipitated fractions was performed to verify comparable immunoprecipitation efficiency, and total cell lysates were analyzed in parallel as input controls. For gain- and loss-of-function analyses, NOZ and GBC-SD cells were transfected with circSMAD4 overexpression plasmids, two independent circSMAD4-targeting siRNAs, or the corresponding controls before immunoprecipitation.*CircRNA sequencing*

High-throughput circRNA sequencing was performed on three pairs of gallbladder cancer (GBC) tissues and matched adjacent non-tumorous tissues. Total RNA was extracted using TRIzol reagent (Invitrogen, USA) and subjected to rRNA depletion and RNase R treatment to enrich for circular RNAs. Library preparation and sequencing were conducted by Novogene Co., Ltd. (Beijing, China), following the manufacturer’s protocols.

CircRNA expression was quantified as counts per million (CPM). Differential expression analysis was performed between GBC tissues and adjacent non-cancerous tissues. For each circRNA, log2 fold change (logFC), average expression level (logCPM), nominal P value, and false discovery rate (FDR)-adjusted q value were calculated. CircRNAs with a nominal P value < 0.05 were considered exploratory candidate differentially expressed circRNAs in the initial discovery analysis. To account for multiple testing, P values were adjusted using the Benjamini-Hochberg procedure, and circRNAs with an FDR-adjusted q value < 0.05 were considered statistically significant after multiple-testing correction. Candidate circRNAs were further evaluated in conjunction with the expression of their corresponding host genes to reduce the potential influence of host-gene transcription on circRNA abundance. Given the limited sample size of the initial sequencing cohort, the circRNA-seq analysis was considered an exploratory discovery screen, and selected candidates were subsequently subjected to independent experimental validation.*CCK-8, transwell and colony formation assays*

CCK-8 cell proliferation assay was carried out by using the CCK-8 Kit (40203ES80, Yeasen, Shanghai, China). 1×10^3^ cells were planted to each well in the 96-well plate and incubated with the CCK-8 reagent for 2 h before obtaining the OD450 values.

For the Transwell assay, 2 x 10^4^ cells were suspended in 200 μl FBS-free DMEM medium and then placed into the upper chamber of transwell inserts (#3422, Corning, State of New York, USA). 600 μl DMEM medium containing 10% FBS was placed into the lower chamber. After 48 h of incubation, the transwell inserts were placed into 10% paraformaldehyde (PFA) for 10 min to fix cells before being stained by the 0.5% crystal violet stain solution (60506ES60, Yeasen, Shanghai, China) for 10 min at RT. Then use the applicator to carefully remove the cells in the upper chamber, and the count of migrated cells was collected by a brightfield microscope (BX43, Olympus, Tokyo, Japan).

1×10^3^ cells were planted to each well in the 6-well plate (or 500 cells in the 12-well plate) and grew for 2 weeks. Then fix the cells in the plate with 10% PFA before staining them with 0.5% crystal violet stain solution at RT for 10 min. The plate was then scanned by a scanner and analyzed by the ImageJ software.

*Subcutaneous mice models*

BALB/c nude mice (PMID: 36549371) with 5~6 weeks old were obtained from Shanghai Model Organisms (<https://www.modelorg.com/>, Shanghai, China). A total of 1×10⁶ wild-type GBC cells or GBC cells stably overexpressing circSMAD4 were subcutaneously injected into the flanks of mice. 7 days later, mice were treated with either 100 μl 5 mg/kg CWI1-2 (Cat. HY-153274, MCE, New Jersey, USA) or vehicle control (DMSO) via tail vein injection once every three days. Tumor growth was monitored using caliper measurements at indicated time points, and were calculated using the equation V = 1/2 × (length × width^2^). After four weeks, mice were euthanized and subcutaneous tumors were subsequently harvested for further analysis. The animal experiments were approved by the Institutional Animal Care and Use Committee of Renji Hospital, Shanghai Jiao Tong University School of Medicine.

*Western blotting*

Cell and tissue total protein was extracted by ice-cold RIPA lysis buffer (Cat. P0013B, Beyotime, Shanghai, China) containing 1 mM PMSF (Cat. ST506, Beyotime, Shanghai, China), and incubated on ice for 10 min followed by centrifuge at 14,000 rcf 4℃ for 10 min. Then, the supernatant of cell lysates was quantified by following the manual of BCA Quantification Kit (Cat. P0010, Beyotime, Shanghai, China). Equal protein samples were resolved in 10% SDS-PAGE gels (Cat. PG112, Epizyme, Shanghai, China) and then transferred to PVDF membranes (Millipore, Massachusetts, USA). PVDF membranes were blocked with QuickBlock™ buffer (Cat. P0240, Beyotime, Shanghai, China) at RT for 10 min. Primary antibodies were incubated at 4℃ overnight, and secondary antibodies were incubated at room temperature (RT) for 1 h. All the primary antibodies used in this study are listed in Supplementary Table 2.

*RNase R and Actinomycin D assays*

GBC cell lines were planted to the 12-well plate. After the cells were adherent to the plate and the confluency reached 70%, they were treated with 5 μg/ml Actinomycin D or DMSO and lysed with RNA Isolation Kit (EZBioscience, Roseville, USA) at indicated time points.

A total of 2 μg of RNA was subjected to RNase R digestion according to the manufacturer’s instructions (Cat. RNR07250, Lucigen, Wisconsin, USA).

*Northern blotting*

Northern blotting was conducted as previously described^2^. A digoxigenin (DIG)-labeled RNA probe complementary to the back-splice junction of circSMAD4 was synthesized using the DIG Northern Starter Kit (Roche, Cat. No. 12039672910), following the manufacturer's instructions. Primer sequences used for probe generation were provided in Supplementary Table 1.

*Immunofluorescence (IF) assay*

GBC cell lines were planted to the 12-well plate at the confluence of 50% and fixed with 100% absolute methanol at RT for 10 min. Cells were than washed with PBS for 2 times before incubation with Immunostaining Permeabilization Buffer with Triton X-100 (Cat. 36317ES60, Yeasen, Shanghai, China) at RT for 10 min. Cells were then washed with PBS for another 2 times and incubated with blocking solution? with 500 μl PBS containing 5% goat serum (Cat. 36119ES10, Yeasen, Shanghai, China) per well at RT for 1 h. Cells were washed with TBST for 3 times and then incubated with primary antibodies at 4℃ overnight. After washing, the cells were incubated them with fluorescent conjugated secondary antibodies. Cell nuclei were stained with DAPI (Cat. P0131, Beyotime, Shanghai, China). Immunofluorescence images were obtained by the confocal fluorescence microscope (TCS SP8 X, Leica, Mannheim, Germany) before analysis with ImageJ software. All the primary antibodies used in this study are listed in Supplementary Table 2.

*Fluorescence in situ hybridization (FISH) and small-molecule FISH (smFISH) assays*

GBC cells were planted to the 24-well plate at the confluency of 50%. Cells were fixed and stained following the manufacturer’s instruction of the FISH Kit (GenePharm, Shanghai, China). The FAM-labeled FISH probe targeting the back-splicing junction site of circSMAD4 was produced by GenePharm Biotech (Shanghai, China).

CircSMAD4 FISH probe sequence: 5'- FAM- UUCAGGAUAACCUUUAUAUAUGCAC -3'.

The small-molecule fluorescence in situ hybridization was performed as previously described with minor modification^3^. Cells or tissues were fixed in 4% paraformaldehyde for 15 min at room temperature, then permeabilized in 70% ice-cold ethanol overnight at -20 °C. After washing with 2× SSC, samples were pre-hybridized at 37 °C for 30 minutes in hybridization buffer (5× SSC, 30% formamide, 9 mM citric acid pH 6.0, 0.1% Tween-20, 10% dextran sulfate, 50 μg/mL heparin, 1× Denhardt’s solution). Hybridization was performed using 2 pmol probes overnight at 37 °C in the same buffer. For signal amplification, 18 pmol hairpins were denatured at 95 °C for 90 seconds and cooled in the dark for 30 min, then applied to samples for overnight incubation at RT. Samples were subsequently washed three times in 2× SSC for 5 min. Nuclei were stained with DAPI (Cat. P0131, Beyotime, Shanghai, China).

FISH and smFISH images were obtained by the confocal fluorescence microscope (TCS SP8 X, Leica, Mannheim, Germany) before analysis with the ImageJ software.

*RNA pulldown*

Cells were lysed with ice-cold Western/IP Lysis Buffer (Cat. P0013, Beyotime, Shanghai, China) containing 1 mM PMSF (Cat. ST506, Beyotime, Shanghai, China) and 200 units/ml Murine RNase Inhibitor Pro (Cat. R303, Vazyme, Jiangsu Province, China), and cell lysates were incubated on ice for 10 min. Biotin-coupled RNAs were synthesized by HuaGene Biotech (Shanghai, China). The biotin-coupled RNA complex was pulled down by incubating the cell lysates with BeyoMag™ Streptavidin Magnetic Beads (Cat. P2151, Beyotime, Shanghai, China) and 2 pmol biotin-labeled RNA probes following the manufacturer’s instructions. The enrichment of circSMAD4 in the captured fractions was confirmed by qRT-PCR. Bound proteins were eluted from magnetic beads using 1× SDS loading buffer (Cat. P0286, Beyotime, Shanghai, China) prior to SDS-PAGE. Eluted proteins from the biotin-labeled RNA complex were subsequently analyzed by western blotting, silver staining, or mass spectrometry analysis. The SDS-PAGE gels were stained by the Fast Silver Staining Kit (Cat. P0017S, Beyotime, Shanghai, China), and the prominent differential protein band was isolated and sent to National Center for Protein Science Shanghai (Shanghai, China) for mass spectrometry analysis. All the biotin-labeled RNA probes are listed in Supplementary Table 1.

*RNA immunoprecipitation (RIP)*

GBC cells were washed twice with ice-cold 1× PBS and lysed in polysome lysis buffer (1000 mM KCl, 50 mM MgCl_2_, 100 mM HEPES-NaOH pH 7, 5% NP-40). Lysates were incubated on ice for 5 min and then snap-frozen at −80 °C for storage or immediate use. Cell lysates were centrifuged at 20,000 × g for 10 min at 4 °C to remove debris. Protein A/G Magnetic Beads (Cat. B23202, Selleck, USA) were washed twice with NT-2 buffer (250 mM Tris-HCl pH 7.4, 750 mM NaCl, 5 mM MgCl_2_, 0.25% NP-40), resuspended, and incubated with 5 μg of target or control antibody at room temperature for 1 h with rotation. Beads were then washed six times with NT-2 buffer to remove unbound antibody. Cleared cell lysate (100 μl, ~2–5 mg total protein) was added to the antibody-bound beads and incubated overnight at 4 °C with rotation. After incubation, the beads were washed six times with ice-cold NT-2 buffer to remove nonspecific binding. Immunoprecipitates were resuspended in Proteinase K buffer (1× NT-2 buffer supplemented with 1% SDS, 1.2 mg/ml Proteinase K) and incubated at 55 °C for 30 min to digest proteins. RNA was extracted by phenol:chloroform:isoamyl alcohol (125:24:1) and purified via additional chloroform extraction. RNA was then precipitated with 50 μl of ammonium acetate 5 M, 15 μl of LiCl 7.5 M, 5 μl glycogen (5 mg/ml), and 850 μl of absolute ethanol at −80 °C for 1 h. Pellets were washed with 80% ethanol, air-dried, and resuspended in RNase-free water. Then, the extracted RNA was subjected to further qRT-PCR analysis. All the primers for qRT-PCR used in RIP assays were listed in Supplementary Table 1. All the antibodies used in this study were listed in Supplementary Table 2.

*Plasmids, siRNA and lentivirus generation*

The circSMAD4 overexpression and empty vector plasmids were synthesized and obtained from Geneseed Biotech (Guangzhou, China). Full-length wild-type IGF2BP2 and its truncated or mutant constructs, each tagged with a 3×FLAG epitope, were cloned into the pCDH-CMV vector (#72265, Addgene, USA, RRID:Addgene_72265) using EcoRI and BamHI restriction sites (Thermo Fisher Scientific) followed by ligation using the ClonExpress II One Step Cloning Kit (Cat. C112, Vazyme, Jiangsu, China). as well as all the other predicted upstream RBPs for circSMAD4 (including U2AF2, HNRNPC, IGF2BP2, etc.). For RNA interference experiments, short hairpin RNAs (shRNAs) were cloned into the pLKO.1-U6-puro vector (#8453, Addgene, USA, RRID:Addgene_8453) via EcoRI and AgeI sites using the above same cloning system. In addition, all siRNAs duplexes were synthesized by HuaGene Biotech (Shanghai, China). The sequences of siRNA and shRNA used in this study were listed in Supplementary Table 1.

Lentiviral particles were produced by co-transfecting HEK-293T cells with the target plasmids, together with packaging plasmids psPAX2 (#12260, Addgene, USA, RRID:Addgene_12260) and pMD2.G (#12259, Addgene, USA, RRID:Addgene_12259). The supernatants containing concentrated lentivirus were collected, filtered, and used to infect gallbladder cancer cell lines (NOZ and GBC-SD). After 48 hours of infection, puromycin (Cat. MA0318, Meilunbio, Liaoning, China) was applied for selection to establish stably shRNA expressing cell lines. The puromycin concentrations used for selection were 2 μg/mL for NOZ cells and 20 μg/mL for GBC-SD cells, respectively.

*Transcriptomic analysis*

GBC-SD cells stably overexpressing circSMAD4 or carrying empty vector controls were subjected to bulk RNA sequencing. Total RNA was extracted using TRIZOL reagent (Cat. 15596018, Thermo Fisher Scientific) following the manufacturer’s protocol and subsequently sent to BGI Genomics (Shenzhen, China) for library preparation, sequencing, and bioinformatic analysis. Libraries were constructed using the NEBNext® Ultra™RNA Library Prep Kit (New England Biolabs, MA, USA), and sequencing was performed on the Illumina platform with paired-end reads. Differential gene expression analysis was conducted using DESeq2 (RRID:SCR_000154), with thresholds set at |log₂ fold change| ≥1 and adjusted p-value <0.05. Gene set enrichment analysis (GSEA) (RRID:SCR_003199) was performed using the GSEA software package (v4.3.2, Broad Institute, Cambridge, MA, USA) to explore the functional relevance of transcriptomic changes between circSMAD4-overexpressing and control GBC-SD cells. The normalized expression matrix was input into GSEA, and analysis was conducted using the hallmark gene sets (h.all.v2023.1.Hs.symbols.gmt) from the Molecular Signatures Database (MSigDB). Enrichment scores were calculated using 1,000 permutations, and gene sets with a false discovery rate (FDR) < 0.25 and nominal p-value < 0.05 were considered significantly enriched.

*Immunohistochemistry staining (IHC)*

Murine and human tissue samples were processed using standard protocols for paraffin embedding and sectioning with a microtome. Paraffin-embedded sections, 3 μm in thickness, were baked at 65 °C overnight. Automated immunohistochemical staining was performed using the BOND-RXm system (Leica, Mount Waverley, Australia) with primary antibodies listed in Supplementary Table 2. Stained sections were visualized using a brightfield microscope (BX43, Olympus, Tokyo, Japan), and image analysis was conducted using ImageJ software (RRID:SCR_003070).

*Ethics*

This study was approved by the Ethics Committee of Renji Hospital, Shanghai Jiao Tong University School of Medicine (Approval No. KY2024-036-C), and conducted in accordance with the ethical principles outlined in the Declaration of Helsinki. Written informed consent was obtained from all participants prior to specimen collection. All animal experiments were reviewed and approved by the Institutional Animal Care and Use Committee (IACUC) of Renji Hospital, Shanghai Jiao Tong University School of Medicine. All procedures involving animals were performed in strict compliance with institutional guidelines and the ARRIVE (Animal Research: Reporting of In Vivo Experiments) guidelines to ensure ethical and humane treatment.

*Statistical analysis*

All experiments were performed in at least three independent biological replicates. Quantitative data were presented as mean ± standard deviation (SD). Statistical analyses were conducted using GraphPad Prism 8.0 (GraphPad Software, La Jolla, CA, USA) and R software (version 4.5.0). Comparisons between two groups were evaluated using the unpaired two-tailed Student’s t-test. Paired two-tailed Student’s t-tests were used for comparisons between matched GBC and adjacent non-cancerous tissues. CircRNA expression heatmap in 3 paired GBC tissues was analyzed in R and compared using Wilcoxon test.

For clinicopathological and survival analyses, circSMAD4 expression was quantified by qRT-PCR in tumor tissues from 65 patients with GBC. The relative expression values were normalized such that the cohort median was set to 1.0. Patients with circSMAD4 expression values ≥1.0 were classified as the high-expression group, whereas those with values <1.0 were classified as the low-expression group. Overall survival analyses were conducted using the Kaplan-–Meier method and compared using the log-rank test, also implemented in R software. The survival analysis was unadjusted, and multivariable Cox proportional hazards regression was not performed; therefore, the observed association between circSMAD4 expression and overall survival was not adjusted for potential clinicopathological confounders. Associations between circSMAD4 expression levels and clinicopathological variables were evaluated using the Chi-square test or Fisher’s exact test, as appropriate.also R. Pearson correlation analysis was used to evaluate correlations between gene expression levels. All statistical tests were two-sided unless otherwise specified. P-values < 0.05 were considered statistically significant. Statistical significance was indicated as follows: P< 0.05 (*), P< 0.01 (**), P< 0.001 (***), and P< 0.0001 (****).

**References**

1.	Wang Z, Wang S, Jia Z, et al. Establishment and characterization of an immortalized epithelial cell line from human gallbladder. *Front Oncol* 2022; **12**: 994087.

2.	Wang X, Shan G. Nonradioactive Northern Blot of circRNAs. *Methods Mol Biol* 2018; **1724**: 135-41.

3.	Choi HMT, Schwarzkopf M, Fornace ME, et al. Third-generation in situ hybridization chain reaction: multiplexed, quantitative, sensitive, versatile, robust. *Development* 2018; **145**(12).

**Supplementary Figures**

**Supplementary Figure 1. Sequence and expression levels of circSMAD4 in GBC.**

(A) Full sequence of circRNA (1435 nt) from circBase (https://circbase.org/).

(B) Representative single molecule fluorescence in situ hybridization (smFISH) images of circSMAD4 in 5 paired gallbladder cancer tissues. GBC tissues were stained with the circSMAD4 probe (green) and DAPI (blue). Scale bar = 20 μm.

(C) Statistical analysis of the circSMAD4 fluorescence in smFISH images of 21 paired GBC and adjacent para-cancer tissues.

Data are shown as the mean ± S.D. The P value was determined by a two-tailed unpaired Student’s t test. **p< 0.01.

**Supplementary Figure 2. Linear transcripts of circSMAD4 do not impact on proliferation or migratory capacity of GBC cells in vitro and in vivo.**

(A) Relative circSMAD4, lin-circSMAD4 and SMAD4 mRNA expression levels in NOZ and GBC-SD cell lines transfected with empty vector (EV) and circSMAD4 overexpression plasmids as indicated using RT-PCR assays, n = 3. Lin-circSMAD4 levels were detected by primers targeting SMAD4 exon 3 while SMAD4 mRNA levels were detected by primers targeting SMAD4 exon 12.

(B) Schematic pattern of circSMAD4 and lin-circSMAD4 overexpression plasmids. (Created in https://BioRender.com)

(C) Relative lin-circSMAD4 expression levels in NOZ and GBC-SD cell lines transfected with empty vector (EV) and circSMAD4 or lin-circSMAD4 overexpression plasmids as indicated using RT-PCR assays, n = 3.

(D) Growth of NOZ and GBC-SD cells transfected with empty vector (EV) and lin-circSMAD4 overexpression plasmids as indicated using CCK-8 assays, n = 3.

(E) Migration of NOZ and GBC-SD cells transfected with EV or lin-circSMAD4 overexpression plasmids as indicated Transwell assays of. Scale bar: 200 μm, n = 3.

(F) Growth of NOZ and GBC-SD cells transfected with lin-circSMAD4 overexpression plasmids using colony formation assays. Scale bar: 5 mm, n = 3.

(G) Relative circSMAD4 and linear transcripts expression in NOZ cells stably overexpressing circSMAD4, n = 3.

(H) Relative linear transcripts expression in NOZ cells stably expressing empty vector (EV) or lin-circSMAD4, n = 3.

(I-J) 1x106 NOZ cells stably expressing EV or lin-circSMAD4 were subcutaneously injected into BALB/c nude mice, and tumors were harvested on post-injection day 28 (I). Tumor growth curves are shown in (J).

Data are shown as the mean ± S.D. The P value was determined by a two-tailed unpaired Student’s t test. ns, not significant *p< 0.05, **p< 0.01, ***p< 0.001, ****p< 0.0001.

**Supplementary Figure 3. IGF2BP2 promotes circSMAD4 stability in GBC cells.**

(A) Spearman’s correlation between SMAD4 mRNA (x axis) and IGF2BP2 mRNA (y axis) expression levels in 30 patient-derived GBC tissues.

(B-C) circSMAD4 levels in NOZ (B) and GBC-SD (C) cells expressing scramble or IGF2BP2 knockdown siRNAs in the presence of 5 μg/ml Actinomycin D via qRT-PCR, n = 3.

Data are shown as the mean ± S.D. The P value was determined by a two-tailed unpaired Student’s t test. **p< 0.01, ***p< 0.001, ***p< 0.0001.

**Fig. S4. The expression of circSMAD4 is dependent on the m6A recognition ability of IGF2BP2 in GBC-SD cells.**

(A) circSMAD4 and SMAD4 mRNA relative expression levels in GBC-SD cells transfected with wild type IGF2BP2 and its various truncated mutants via qRT-PCR analysis, n = 3.

(B) circSMAD4 and SMAD4 mRNA relative expression levels in GBC-SD cells after being transfected with wild type IGF2BP2 and its various mutants via qRT-PCR analysis, n = 3.

(C) circSMAD4 and SMAD4 mRNA relative expression levels in GBC-SD cells after co-transfection with indicated siRNA and mutant plasmids via qRT-PCR analysis, n = 3.

(D-E) Prediction of m6A modification sites in intron 1 of SMAD4 pre-mRNA using SRAMP.

(F-G) Prediction of m6A modification sites in intron 10 of SMAD4 pre-mRNA using SRAMP.

(H) m6A modification sites in the circSMAD4pre-mRNA transcript in GBC-SD cells via MeRIP assay using anti-m6A antibody, n = 3.

(I) Putative binding sites of IGF2BP2 in the circSMAD4pre-mRNA transcript in GBC-SD cells via RIP assay using anti-IGF2BP2 antibody, n = 3.

(J) Failure of interaction between IGF2BP2 and the m6A modifications sites in the circSMAD4 pre-mRNA transcript in cells expressing KH3-4 domains of IGF2BP2 via RIP assay using anti-Flag antibody, n = 3.

Data are shown as the mean ± S.D. The *P* value was determined by a two-tailed unpaired Student’s t test. ns, not significant **p*< 0.05, ***p*< 0.01, ****p*< 0.001, *****p*< 0.0001.

**Supplementary Figure 5. Evaluation of the translational potential and miRNA-binding ability of circSMAD4.**

(A) Illustration of the putative open reading frame (ORF) of circSMAD4.

(B) circSMAD4 and SMAD4 mRNA overexpression plasmids carrying 3xFlag-tag immediately before the stop codon.

(C) Expression of circSMAD4 or SMAD4 in cells expressing above 3xFlag tag (B) in Western blotting using anti-Flag antibody.

(D-E) Ribosome profiling analysis of circSMAD4 translational potential. Cellular lysates were fractionated according to ribosome association (D), and the distribution of circSMAD4 across the collected fractions was quantified by qRT-PCR, with ACTB mRNA included as a reference transcript, n = 3 (E). LMW, low molecular weight; HMW: high molecular weight.

(F) Interaction between circSMAD4 and Ago-2 using RIP assay followed by qRT-PCR. First column represented a positive control that used 10% RNA sample of equal loading samples, n = 3.

Data are shown as the mean ± S.D. The *P* value was determined by a two-tailed unpaired Student’s t test. ns, not significant **p*< 0.05, ***p*< 0.01, ****p*< 0.001.

**Supplementary Figure 6. CWI1-2 inhibits GBC development induced by circSMAD4 in vivo and in vitro.**

(A) Growth in NOZ and GBC-SD cells overexpressing circSMAD4 in the presence of DMSO or 2 μM CWI1-2 via colony formation assays. Scale bar: 5 mm, n = 3.

(B) Xenografted tumors removed from mice carrying GBC-SD cells stably overexpressing empty vector (EV) and circSMAD4 on day 25 after treatment with DMSO or 5 mg/kg CWI1-2.

(C) The curves of tumor growth.

(D) Representative images of H&E staining and IHC for expression levels of IGF2BP2, Ki67 and p-STAT3 proteins. Scale bar: 100 μm.

Data are shown as the mean ± S.D. The *P* value was determined by a two-tailed unpaired Student’s t test. *****p*< 0.0001.
